# METTL7A Downregulation Drives SLC1A5‐Mediated Glutamine Competition to Promote Tumor Proliferation and Suppress CD8^+^ T Cell Immunity in Gastric Cancer

**DOI:** 10.1002/advs.76963

**Published:** 2026-08-03

**Authors:** Mingjun Sun, Shuwei Dang, Dazhi Zhou, Lixian Ding, Xun Sun, Wei Suo, Jirui Niu, Qingzhu Yang, Zhicheng Zhang, Yanyan Sun, Jinxing Li, Zhongsheng Chen, Tao Ban, Yingji Wang, Tianzhu Li, Ming Liu, Guodong Li

**Affiliations:** ^1^ Department of General Surgery The Fourth Affiliated Hospital of Harbin Medical University Harbin China; ^2^ Heilongjiang Provincial Key Laboratory of Gastrointestinal Surgery and Nutrition Metabolism Harbin China; ^3^ Future Medical Laboratory The Second Affiliated Hospital of Harbin Medical University Harbin China; ^4^ Department of Vascular Surgery The First Affiliated Hospital of Fujian Medical University Fuzhou China; ^5^ Department of Vascular Surgery National Regional Medical Center Binhai Campus of the First Affiliated Hospital Fujian Medical University Fuzhou China; ^6^ College of Life Science and Agriculture Forestry Qiqihar University Qiqihar China; ^7^ Department of General Surgery Harbin Fourth Hospital Harbin China; ^8^ Department of Pharmacology College of Pharmacy Harbin Medical University Harbin China; ^9^ Department of Inorganic Chemistry and Physical Chemistry College of Pharmacy Harbin Medical University Harbin China; ^10^ State Key Laboratory of Neurology and Oncology Drug Development Nanjing China

**Keywords:** CD8^+^ T cells, glutamine competition, METTL7A, SLC1A5

## Abstract

Tumor metabolic dysregulation is a critical determinant of tumor progression and response to immunotherapy. Aberrant glutamine metabolism is a hallmark of gastric cancer (GC). However, beyond fueling GC cell anabolism, its role in remodeling the immunosuppressive tumor microenvironment remains poorly understood. Here, we show that GC cells overexpress solute carrier family 1 member 5 (SLC1A5) to drive glutamine accumulation, which not only promotes their own proliferation but also reduces glutamine availability to CD8^+^ T cells, thereby suppressing antitumor immunity. These dual effects cooperatively drive GC progression. Mechanistically, loss of methyltransferase‐like protein 7A (METTL7A) stabilizes SLC1A5 mRNA by reducing its m6A modification. Concurrently, METTL7A deficiency increased N‐glycosyltransferase β‐1,4‐galactosyltransferase 5 (B4GALT5) expression. B4GALT5 stabilizes SLC1A5 via N‐glycosylation at the N212 site, which blocks K48‐linked polyubiquitination and proteasomal degradation. We identify the natural flavonoid luteolin as an agent that upregulates METTL7A expression, which subsequently downregulates SLC1A5 expression and inhibits GC progression. Furthermore, luteolin significantly enhances the efficacy of anti‐PD‐1 therapy in GC. Collectively, our findings reveal that SLC1A5‐mediated glutamine competition drives both tumor cell proliferation and immune evasion in GC, and suggest that targeting the METTL7A/SLC1A5 axis may represent a promising therapeutic strategy.

## Graphical abstract

GC cells enhance glutamine accumulation by upregulating SLC1A5 expression. This upregulation not only boosts GC cell proliferation, but also outcompetes CD8^+^ T cells for glutamine and suppresses their antitumor immunity. Mechanistically, METTL7A deficiency in GC induces SLC1A5 overexpression via an m6A‐dependent pathway and N‐glycosylation‐ubiquitination antagonism. Luteolin targets the METTL7A/SLC1A5 axis to inhibit GC progression and enhances anti‐PD‐1 therapy efficacy.

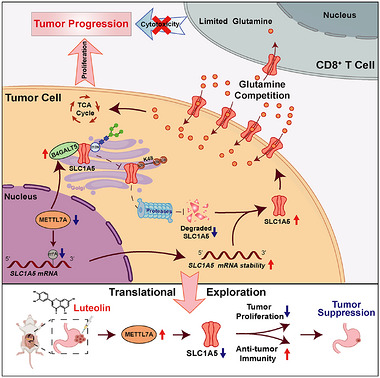

## 1 Introduction

Gastric cancer (GC) remains a major global health burden, ranking as the fifth leading cancer worldwide in both incidence and mortality [1]. While locally advanced GC is potentially curable, patients with advanced or metastatic GC have a poor prognosis, with a median overall survival of less than one year [2]. Immunotherapy for GC has made significant progress, showing notable efficacy particularly in patients with microsatellite instability or deficient mismatch repair subtypes [1, 3, 4]. However, these subtypes collectively account for only approximately 5% of advanced GC cases, severely limiting the overall benefit of current immunotherapy [2, 5]. Therefore, identifying novel therapeutic targets, developing more effective combination therapies, and expanding the clinical applicability of immunotherapy are critical to improve outcomes for patients with GC.

Glutamine is a critical yet contested amino acid within the tumor microenvironment (TME), as it is essential for both the growth of tumor cells and the functions of CD8^+^ T cells [6, 7, 8]. Previous research on glutamine metabolism has primarily focused on GC cell‐intrinsic mechanisms. For example, the lncRNA NR_033928 enhances glutamine metabolism, thereby promoting proliferation and suppressing apoptosis in GC cells [9, 10, 11]. However, the cell‐centric mechanisms are incomplete without consideration of the complex interactions within the TME. Emerging evidence reveals that tumor cells can overexpress specific solute carriers to actively deprive CD8^+^ T cells of key amino acids. Representative examples include SLC3A2‐driven arginine competition in hepatocellular carcinoma and SLC6A6‐mediated taurine competition in GC [12, 13]. These findings suggest that amino acid competition via solute carrier (SLC) family overexpression may represent a common tumor immune evasion strategy. Solute carrier family 1 member 5 (SLC1A5), the major cellular glutamine transporter, is frequently overexpressed in tumors and promotes tumor progression by enhancing glutamine uptake [14, 15, 16]. To date, it remains unclear whether glutamine competition exists between GC cells and CD8^+^ T cells, and the precise role of SLC1A5 in this context also remains undefined.

The malignant progression of GC is driven by intercellular crosstalk within the TME and multilayered oncogenic regulatory networks. As the most prevalent internal RNA modification in eukaryotes, N6‐methyladenosine (m6A) exerts critical effects on tumorigenesis by modulating RNA stability, translation, and splicing [17]. Although m6A modification has been reported for SLC1A5 mRNA, its impact on SLC1A5 expression appears to be highly disease‐specific [18, 19, 20, 21], and its function and underlying mechanisms in GC remain elusive. Furthermore, SLC1A5 is known to undergo N‑glycosylation [22]. However, the biological function and upstream regulatory mechanisms of this modification remain largely unknown in GC. Clarifying the upstream regulation of SLC1A5 at both post‐transcriptional and post‐translational levels is essential to advance the understanding of GC pathogenesis and expand therapeutic strategies.

In this study, we demonstrate that GC cells drive glutamine competition by overexpressing SLC1A5, which plays a dual role in promoting GC cell proliferation and impairing CD8^+^ T cell antitumor immunity. Mechanistically, METTL7A coordinately suppresses SLC1A5 expression in GC cells through an m6A‐dependent pathway and antagonistic N‐glycosylation–ubiquitination axis. Targeting the METTL7A/SLC1A5 axis with the natural flavonoid luteolin may represent a potential therapeutic strategy to suppress GC progression and enhance anti‑PD‑1 efficacy. Collectively, our findings reveal that SLC1A5‐mediated glutamine competition drives both GC cell proliferation and immune evasion, providing a strong mechanistic rationale for therapeutically targeting the METTL7A/SLC1A5 axis.

## 2 Results

### 2.1 High SLC1A5 Expression Correlates With Poor Prognosis in GC Patients and Promotes GC Cell Proliferation via Glutamine Accumulation

**FIGURE 1:**
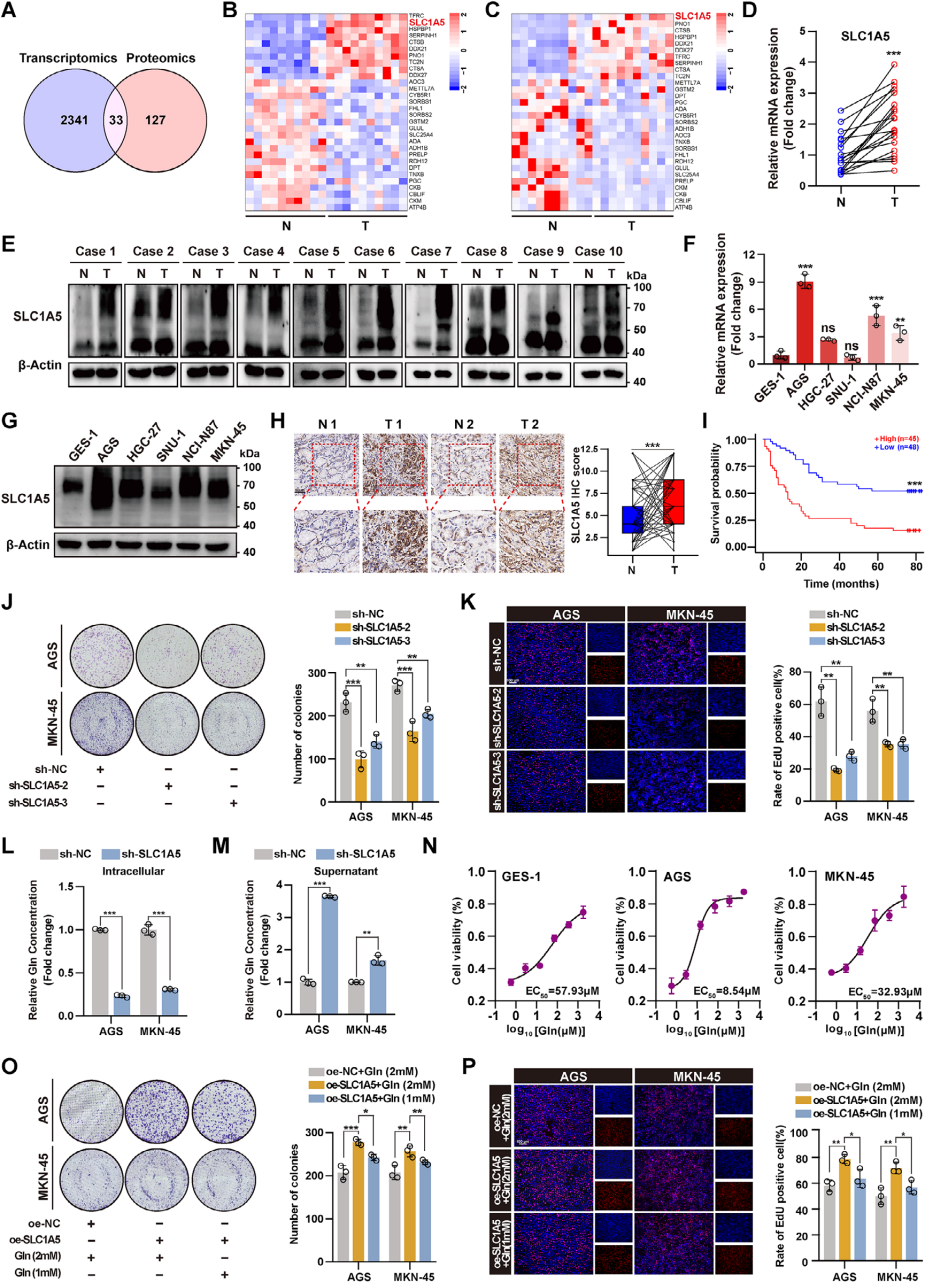
High SLC1A5 expression is associated with poor prognosis and promotes proliferation via glutamine accumulation in GC. (A) Venn diagram representing the overlap between DEGs (transcriptomic) and DEPs (proteomics). (B, C) Heatmaps displaying the expression patterns of the 30 hub genes at the transcriptomic (B) and proteomic (C) levels. (D, E) qRT‐PCR analysis (D) and Western blot analysis (E) of SLC1A5 expression in paired GC and adjacent noncancerous tissues. (F, G) qRT‐PCR analysis (F) and Western blot analysis (G) of SLC1A5 expression in GES‐1 and GC cells. (H) Representative immunohistochemistry (IHC) images and quantification of SLC1A5 in GC tissues and paired adjacent noncancerous tissues from the in‐house cohort. Scale bar: 50 µm. (I) Kaplan–Meier survival curves according to SLC1A5 expression in the in‐house GC cohort. (J, K) Colony formation (J) and EdU staining (K) assays in SLC1A5 knockdown AGS and MKN‐45 cells. n = 3. Scale bars: 100 µm. (L, M) Intracellular glutamine concentration (L) and glutamine concentration of the cell culture supernatant (M) in AGS and MKN‐45 cells after SLC1A5 knockdown. n = 3. (N) Viability effect of glutamine in GES‐1, AGS, and MKN‐45 cells. Half‐maximal effective concentration (EC_50_) values are indicated. n = 3. (O, P) Colony formation (O) and EdU staining (P) assays of SLC1A5‐overexpressing cells under normal (2 mM) or reduced (1 mM) glutamine conditions. n = 3. Scale bars: 100 µm.

To investigate key genes underlying GC progression, we performed transcriptomic and proteomic sequencing of 10 matched pairs of GC and adjacent noncancerous tissues. We identified 2374 differentially expressed genes (DEGs) from the transcriptomic dataset and 160 differentially expressed proteins (DEPs) from the proteomic dataset (|log_2_FC| > 1, *p* < 0.05). The overlap between the 2374 DEGs and 160 DEPs revealed 33 genes with concordant differential expression (Figure 1A), with 30 hub genes exhibiting consistent regulation at both levels (10 upregulated and 20 downregulated). Subsequently, we performed KEGG and GO analysis on these 30 hub genes. The KEGG analysis revealed significant enrichment in several metabolic pathways, including tyrosine metabolism, retinol metabolism, and phenylalanine metabolism. Similarly, the GO analysis demonstrated substantial enrichment across metabolic functions (Figure S1A, B). Separate gene expression heatmap analyses were conducted for these 30 hub genes on the transcriptomic and proteomic datasets. We observed that SLC1A5 was markedly increased, representing the second most highly expressed gene in the transcriptomic dataset and the most highly expressed protein in the proteomic dataset (Figure 1B, C). This significant upregulation of SLC1A5 was validated at both the mRNA and protein levels in GC tissues compared to adjacent noncancerous tissues (Figure 1D, E). Furthermore, SLC1A5 expression was higher in most human GC cell lines than in gastric epithelial cell line‐1 (GES‐1) (Figure 1F, G).

In order to establish the clinical relevance of SLC1A5 in GC progression, we assembled an in‐house cohort of 93 GC patients. Immunohistochemistry (IHC) analysis confirmed that SLC1A5 protein levels were significantly higher in GC tissues than in adjacent noncancerous tissues (Figure 1H). Moreover, SLC1A5 expression was positively associated with more advanced AJCC stage, pathological grade, invasion depth, and lymph node metastasis (Figure S1C–F). High SLC1A5 expression also served as a predictor of poor prognosis in GC patients (Figure 1I). The results from the analysis of TCGA and GEO datasets were consistent with our findings (Figure S1G–J).

To determine the functional role of SLC1A5 in GC growth, we established AGS and MKN‐45 cell lines with stable knockdown of SLC1A5 (Figure S2A, B). Results from the Colony formation, EdU staining, and CCK‐8 assays demonstrated that SLC1A5 knockdown markedly inhibited cell proliferation in both AGS and MKN‐45 cells (Figure 1J, K and Figure S2C). These findings suggest that SLC1A5 facilitates GC cell proliferation. Given that SLC1A5 serves as a key glutamine transporter in cells and that glutamine critically supports the highly proliferative state of tumor cells [23], we hypothesized that SLC1A5 promotes GC progression mainly through facilitating glutamine accumulation to support cell proliferation. SLC1A5 knockdown increased glutamine concentration in the culture medium and decreased intracellular glutamine levels (Figure 1L, M), indicating that SLC1A5 enhances glutamine accumulation in GC cells. To assess the impact of glutamine on GC cells, we exposed GES‐1, AGS, and MKN‐45 cells to a range of glutamine concentrations. The lower half‐maximal effective concentration (EC_50_) in AGS and MKN‐45 cells, compared with that in GES‐1 cells, indicated heightened dependence and sensitivity to glutamine for proliferation in GC cells (Figure 1N). Furthermore, elevating the concentration of glutamine in the culture medium from 2 mM to 4 mM significantly enhanced the proliferative capacity of GC cells (Figure S2D–F). These results demonstrate that glutamine serves as a critical nutrient for GC cell proliferation. Next, we successfully overexpressed SLC1A5 in AGS and MKN‐45 cells (Figure S2G, H). SLC1A5 overexpression in GC cells markedly promoted proliferation under normal culture conditions, which was rescued by culturing in glutamine‐reduced medium (Figure 1O, P and Figure S2I). To further confirm that SLC1A5 promotes GC cell proliferation via glutamine transport, we used the SLC1A5‐specific inhibitor V‐9302, a competitive small‑molecule inhibitor of SLC1A5‑mediated glutamine uptake that does not alter SLC1A5 protein expression [24]. Consistently, V‐9302 treatment significantly reduced the proliferation of AGS and MKN‐45 cells (Figure S2J–L). Collectively, these results demonstrate that SLC1A5 is highly expressed in GC and that its expression is associated with poor prognosis in GC patients. Furthermore, SLC1A5 promotes GC cell proliferation by enhancing glutamine accumulation.

### 2.2 GC Cells Inhibit CD8^+^ T Cell Antitumor Immunity via Glutamine Competition

**FIGURE 2:**
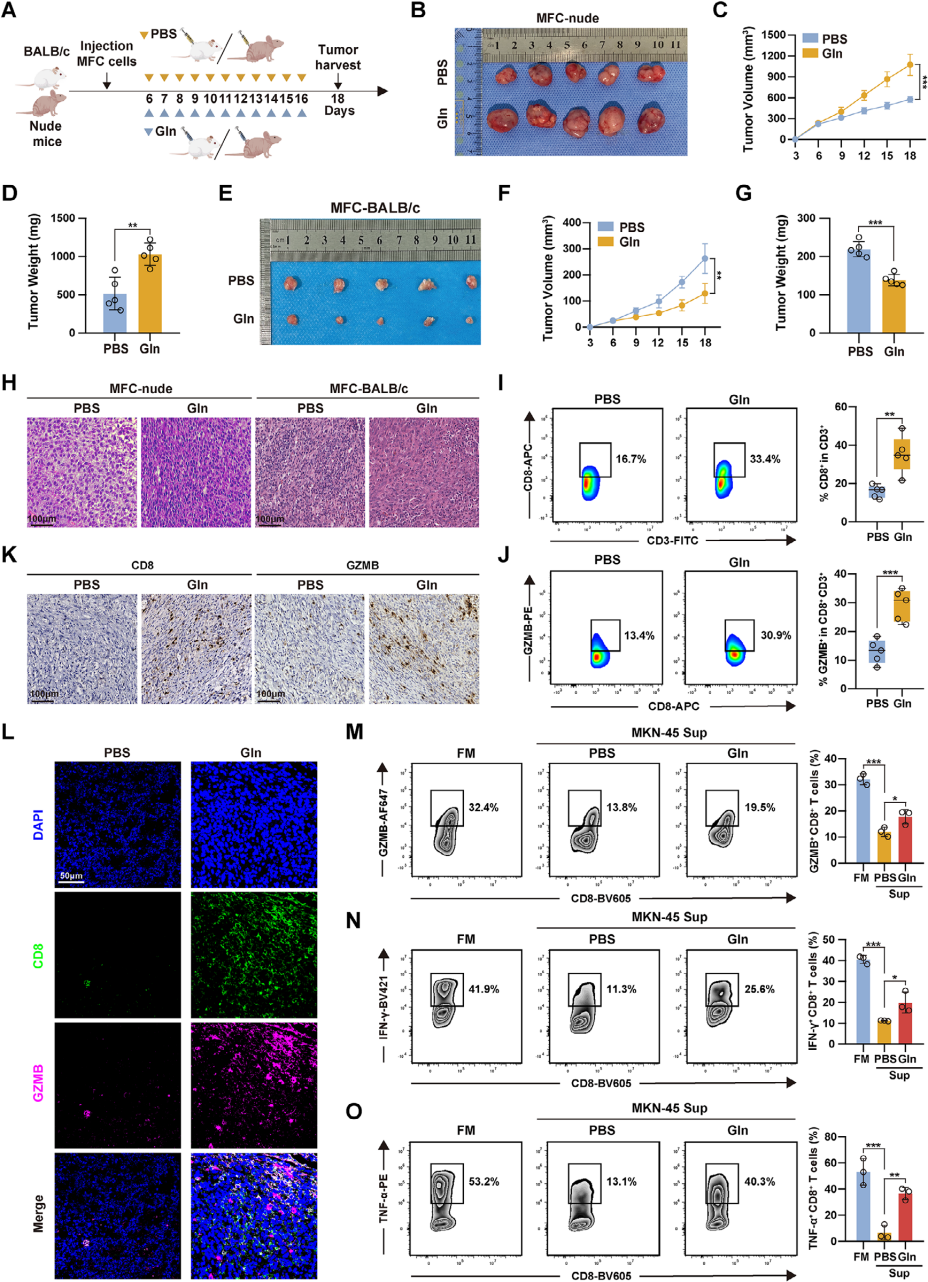
Glutamine exerts opposing effects on tumor growth by restoring CD8^+^ T cell function. (A) Schematic diagram of the drug administration plan in the MFC xenograft model in BALB/c‐nu/nu (nude) and BALB/c mice. (B–D) Representative images (B), tumor growth curve (C), and tumor weights (D) of the subcutaneous xenograft model treated with PBS or glutamine in BALB/c‐nu/nu mice. n = 5. (E–G) Representative images (E), tumor growth curve (F), and tumor weights (G) of the subcutaneous xenograft model treated with PBS or glutamine in BALB/c mice. n = 5. (H) Representative H&E staining of tumor sections from BALB/c‐nu/nu and BALB/c mice. Scale bars: 100 µm. (I, J) Flow cytometry analysis of tumor‐infiltrating CD8^+^ T cells (I) and Granzyme B^+^ (GZMB)^+^ CD8^+^ T cells (J) treated with PBS or glutamine in BALB/c mice. n = 5. (K) Representative IHC images showing CD8 and GZMB staining in PBS‐or glutamine‐treated tumors in BALB/c mice. Scale bars: 100 µm. (L) Representative immunofluorescence staining images of tumor sections from BALB/c mice, showing the expression of CD8 and GZMB. Scale bars: 50 µm. (M–O) Flow cytometry analysis showing the production of GZMB (M), IFN‐γ (N), and TNF‐α (O) by activated human CD8^+^ T cells cultured in normal medium, MKN‐45 cell‐conditioned medium (CM), or CM supplemented with 2 mM glutamine. n = 3.

Our preliminary data demonstrated that glutamine enhanced the proliferative capacity of GC cells. Similarly, existing research has demonstrated that glutamine fosters aggressive phenotypes in tumor cells [25]. Paradoxically, a previous report showed that glutamine exerts its tumor suppressive effect by activating the antitumor immunity of CD8^+^ T cells [26]. To evaluate the in vivo effects of glutamine, we utilized MFC subcutaneous tumor models in both BALB/c‐nu/nu (nude) mice and BALB/c mice, treating them with daily intratumoral injections of either PBS or glutamine (Figure 2A). Consistent with our in vitro findings, intratumoral injection of glutamine in nude mice resulted in a significant increase in both the volume and weight of tumors compared to the PBS‐injected group (Figure 2B–D). In stark contrast, glutamine treatment significantly suppressed the tumor volume and weight in BALB/c mice (Figure 2E–G). H&E staining revealed that the glutamine treatment group in nude mice exhibited nuclear crowding and structural disorganization. Conversely, those in BALB/c mice showed an opposite pattern (Figure 2H). These results demonstrate an opposite effect of glutamine treatment on tumor growth between BALB/c‐nu/nu mice and BALB/c mice.

The nude mice are immunodeficient due to thymic deficiency and the resulting lack of T cells, in contrast to immunocompetent BALB/c mice. In addition, as an essential nutrient, glutamine was reported to enhance CD8^+^ T cell function [26]. Therefore, glutamine treatment may suppress tumor growth in immunocompetent mice by restoring CD8^+^ T cell function. To test this possibility, we performed flow cytometry and IHC staining, which showed that glutamine treatment increased intratumoral infiltration of CD8^+^ T cells and Granzyme B (GZMB)^+^ CD8^+^ T cells in immunocompetent mice (Figure 2I–K). Similarly, immunofluorescence staining revealed that glutamine treatment significantly increased intratumoral CD8^+^ T cell infiltration and the proportion of GZMB^+^ CD8^+^ T cells (Figure 2L). Compared to tumors treated with PBS, those treated with glutamine exhibited increased production of cytotoxic effectors and cytokines, including GZMB, IFN‐γ, and TNF‐α (Figure S3A–C). These findings collectively indicate that exogenous glutamine supplementation restores CD8^+^ T cell antitumor function, thereby suppressing GC progression.

Previous studies have demonstrated that tumor cells compete with immune cells for nutrients including lysine, taurine, and glucose, resulting in impaired immune cell function [12, 13, 27]. We therefore hypothesized that glutamine may be limited within the GC microenvironment, as GC cells outcompete CD8^+^ T cells for endogenous glutamine and thereby impair their function. This could also explain why glutamine supplementation suppresses tumor growth in immunocompetent mice. To validate this hypothesis, we first examined glutamine levels within the GC microenvironment. We quantified glutamine levels in five pairs of human GC tissues and matched adjacent normal tissues. Glutamine content was significantly reduced in tumor tissues compared with paired normal tissues (Figure S3D). We further established subcutaneous GC xenograft models in mice and collected tumor interstitial fluid (TIF) and paired plasma samples. Glutamine concentration was notably lower in TIF than in plasma (Figure S3E). These data suggest that glutamine deficiency occurs in the TME. Then, we isolated human CD8^+^ T cells from peripheral blood mononuclear cells (PBMCs) obtained from healthy volunteers and activated them with anti‑CD3/CD28 stimulation. When these activated CD8^+^ T cells were co‑cultured with MKN‐45 cells, their intracellular glutamine level and the percentage of Ki67^+^ CD8^+^ T cells were significantly reduced (Figure S3F, G). To exclude the influence of direct cell contact, we cultured activated CD8^+^ T cells with MKN‐45 cell supernatant. The supernatant suppressed production of cytotoxic effectors and cytokines in CD8^+^ T cells, while supplementation with 2 mM glutamine restored CD8^+^ T cell function (Figure 2M–O). In summary, GC cells outcompete CD8^+^ T cells for glutamine in the TME, thereby impairing CD8^+^ T cell antitumor immunity.

### 2.3 GC Cells Outcompete CD8^+^ T Cells for Glutamine by Overexpressing SLC1A5

**FIGURE 3:**
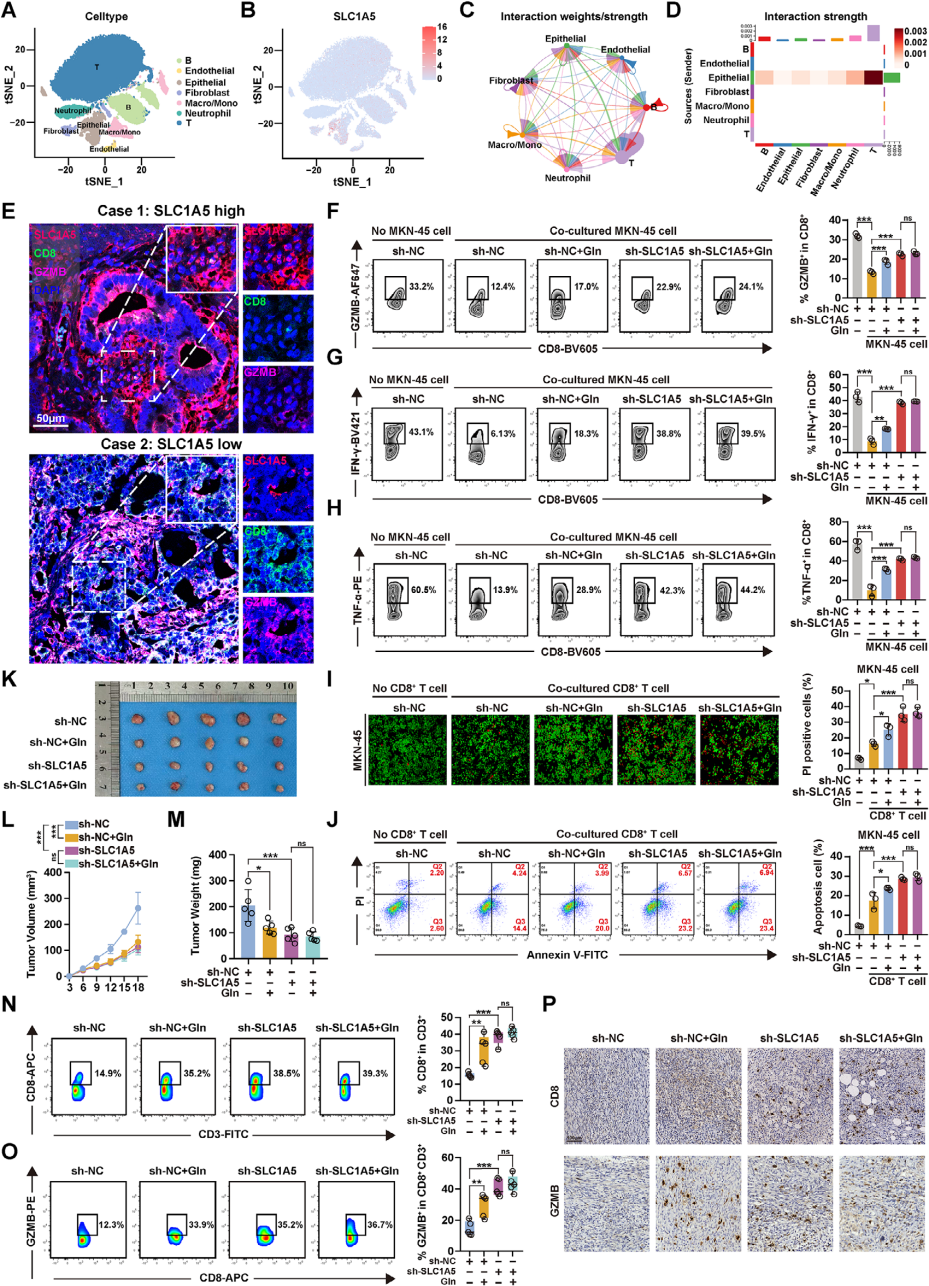
GC cells inhibit CD8^+^ T cell antitumor immunity through SLC1A5‐mediated glutamine competition. (A) t‐SNE plot showing cell clustering of GC single‐cell RNA‐seq data (GSE163558; GSE184198). (B) t‐SNE plot showing SLC1A5 expression across all cell types. (C) Network plot of interaction weights/strength among different cell types. (D) Heatmap of interaction strength between epithelial cells and other cell types. (E) Representative immunofluorescence staining images of CD8^+^ T cells and GZMB^+^ CD8^+^ T cells in human GC tissues with high or low SLC1A5 expression. Scale bars: 50 µm. (F–H) Flow cytometric analysis of human CD8^+^ T cells positive for GZMB (F), IFN‐γ (G), or TNF‐α (H) across different treatment groups. n = 3. (I, J) Viability of MKN‐45 cells assessed by Calcein‑AM/PI staining (I) and flow cytometry (J) across different treatment groups. n = 3. (K–M) Representative images (K), tumor growth curves (L), and tumor weights (M) of sh‐NC or SLC1A5 knockdown MFC tumors in BALB/c mice with or without intratumoral glutamine treatment. n = 5. (N, O) Flow cytometry analysis of tumor‐infiltrating CD8^+^ T cells (N) and GZMB^+^ CD8^+^ T cells (O) from sh‐NC or SLC1A5 knockdown MFC tumors in BALB/c mice with or without intratumoral glutamine treatment. n = 5. (P) Representative IHC images showing CD8 and GZMB staining from sh‐NC or SLC1A5 knockdown MFC tumors in BALB/c mice with or without intratumoral glutamine treatment. Scale bars: 100 µm.

Our above data demonstrated that GC cells enhanced glutamine accumulation by upregulating SLC1A5. Thus, we proposed that GC cells impaired CD8^+^ T cell function through SLC1A5‐mediated glutamine competition. To test this hypothesis, we first performed t‐SNE‐based cell clustering and cell‐type annotation on the GC single‐cell RNA sequencing data (GSE163558; GSE184198) (Figure 3A and Figure S4A). Notably, within the epithelial cell population, SLC1A5 expression was predominantly enriched in tumor epithelial cells (Figure 3B and Figure S4B, C). Spatial transcriptomic analysis further confirmed that SLC1A5 expression was highly enriched in tumor regions (Figure S4D). Next, we explored cell‐cell communication networks within the GC microenvironment using cell interaction analysis. We observed a strong interaction between tumor epithelial cells and T cells in GC samples. (Figure 3C, D and Figure S4E). Based on this finding, we next examined whether SLC1A5 expression correlates with CD8^+^ T cell infiltration in human GC tissues. Immunofluorescence staining revealed that, compared to human GC tissues with low SLC1A5 levels, those with high SLC1A5 expression had significantly reduced intratumoral infiltration of CD8^+^ T cells and GZMB^+^ CD8^+^ T cells (Figure 3E). Subsequently, we compared SLC1A5 expression in activated CD8^+^ T cells and GC cells, and found that the expression level of SLC1A5 was significantly higher in human GC cells than in human CD8^+^ T cells (Figure S4F). To further verify that GC cells outcompete CD8^+^ T cells for glutamine via SLC1A5, we performed a co‑culture assay using MKN‐45 cells and activated CD8^+^ T cells. When SLC1A5 was knocked down in MKN‐45 cells within the co‑culture system, the intracellular glutamine content in these MKN‐45 cells significantly decreased. Meanwhile, CD8^+^ T cells co‐cultured with SLC1A5 knockdown MKN‐45 cells showed higher intracellular glutamine levels and a higher percentage of Ki67^+^ CD8^+^ T cells than those co‑cultured with control MKN‑45 cells (Figure S3F, G). Collectively, these data suggest that GC cells can outcompete CD8^+^ T cells for glutamine through SLC1A5.

To further validate that SLC1A5‐mediated glutamine competition by GC cells inhibits CD8^+^ T cell function, we performed flow cytometry. The results revealed that co‐culture with MKN‐45 cells reduced the function of CD8^+^ T cells. This functional impairment was rescued either by the addition of glutamine or by knocking down SLC1A5 in the MKN‐45 cells. Furthermore, when co‐cultured with SLC1A5 knockdown MKN‐45 cells, CD8^+^ T cell functions were no longer significantly improved by the addition of glutamine (Figure 3F–H). These suggest that SLC1A5 knockdown alone relieves glutamine competition and restores CD8^+^ T cell function. Similarly, both glutamine supplementation and SLC1A5 knockdown in MKN‐45 cells suppressed the viability of the co‐cultured GC cells. In SLC1A5 knockdown MKN‐45 cells, glutamine supplementation showed no measurable effect on GC cell viability during co‐culture with CD8^+^ T cells (Figure 3I, J and Figure S4G, H).

To determine whether this mechanism operates in vivo, we generated MFC cells with stable SLC1A5 knockdown (Figure S4I, J), which were engrafted into BALB/c mice with or without glutamine treatment. Glutamine treatment or SLC1A5 knockdown suppressed tumor growth. However, intratumoral glutamine injection in SLC1A5 knockdown tumors exerted no measurable effect on tumor growth (Figure 3K–M and Figure S4K). Moreover, either glutamine treatment or SLC1A5 knockdown significantly increased the infiltration of CD8^+^ T cells and GZMB^+^ CD8^+^ T cells. In the SLC1A5 knockdown tumor, intratumoral glutamine supplementation did not further enhance CD8^+^ T cell infiltration or function (Figure 3N–P and Figure S4L, M). Taken together, SLC1A5 upregulation in GC cells drives glutamine accumulation to support GC cell proliferation while crippling CD8^+^ T cell function through glutamine competition, ultimately synergizing to promote GC progression.

### 2.4 METTL7A Inhibits GC Cell Proliferation and Enhances CD8^+^ T Cell Antitumor Immunity by Accelerating SLC1A5 mRNA Degradation in an m6A‐Dependent Manner

**FIGURE 4:**
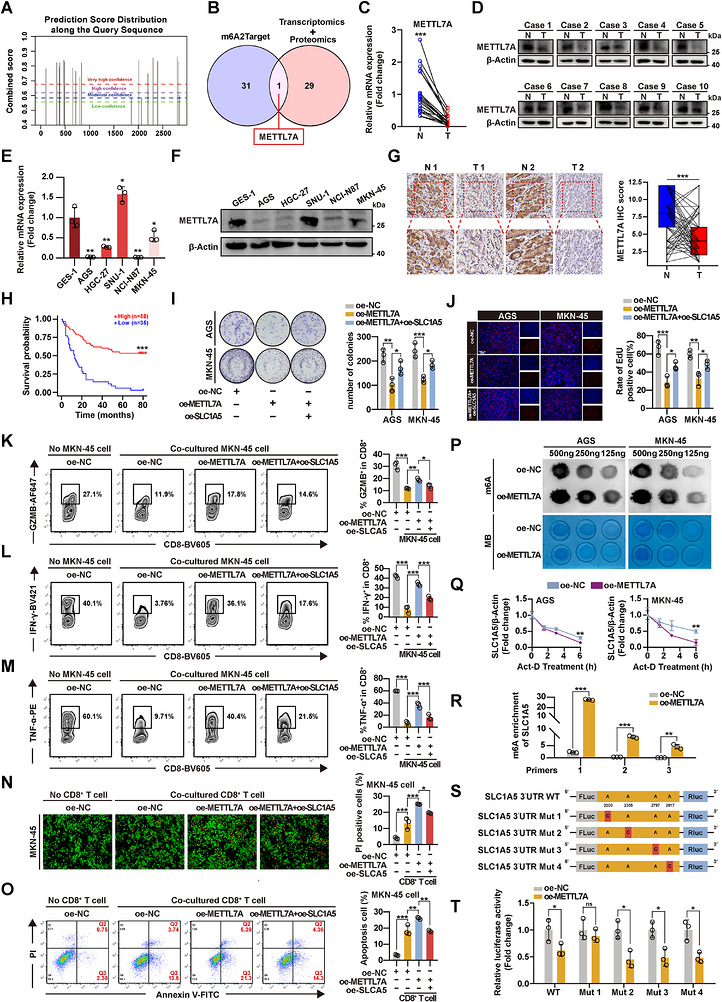
METTL7A suppresses SLC1A5 expression via m6A modification to inhibit GC cell proliferation and restore CD8^+^ T cell antitumor immunity. (A) Predicted m6A modification sites on SLC1A5 mRNA from the SRAMP database. (B) Venn diagram identifying METTL7A as the sole overlapping gene between the m6A2Target database and multi‐omics analysis. (C, D) qRT‐PCR analysis (C) and Western blot analysis (D) of METTL7A expression in paired GC and adjacent noncancerous tissues. (E, F) qRT‐PCR analysis (E) and Western blot analysis (F) of METTL7A expression in GES‐1 and GC cells. n = 3. (G) Representative IHC images and quantitative analysis of METTL7A protein expression. Scale bar: 50 µm. (H) Kaplan–Meier survival curves according to METTL7A expression in the in‐house GC cohort. (I, J) Colony formation (I) and EdU staining (J) assays were conducted in AGS and MKN‐45 cells as indicated. Scale bars: 100 µm. n = 3. (K–M) Flow cytometric analysis of human CD8^+^ T cells positive for GZMB (K), IFN‐γ (L), or TNF‐α (M) across different treatment groups. n = 3. (N, O) Viability of MKN‐45 cells assessed by Calcein‑AM/PI staining (N) and flow cytometry (O) across different treatment groups. n = 3. (P) Dot blot assay was conducted in METTL7A overexpression AGS and MKN‐45 cells. (Q) RNA stability assay was conducted in METTL7A overexpression AGS and MKN‐45 cells. n = 3. (R) MeRIP‐qPCR analysis of m6A enrichment across 3′ UTR of SLC1A5 mRNA in oe‐NC and METTL7A‐overexpressing cells. n = 3. (S) Schematic of the luciferase reporter constructs containing the wild‐type (WT) or mutant (Mut1–4) SLC1A5 3′ UTR. (T) Luciferase activity of reporters containing the WT or mutant (Mut1–4) SLC1A5 3′ UTR in oe‐NC versus METTL7A‐overexpressing cells. n = 3.

We sought to elucidate the mechanisms underlying the SLC1A5 overexpression in GC. N6‐methyladenosine (m6A) is the most prevalent eukaryotic RNA modification and promotes tumor progression through diverse mechanisms such as regulating RNA stability, subcellular localization, and trafficking [17]. Using the SRAMP database, we predicted 19 potential m6A modification sites on SLC1A5 mRNA, including four very high confidence sites in the 3′ untranslated region (3′ UTR) (Figure 4A). We therefore aimed to identify the m6A regulator that regulates SLC1A5. Next, we intersected the list of m6A‐related genes from the m6A2Target database with 30 hub genes from clinical tissue transcriptomics and proteomics. METTL7A was identified as the sole overlapping gene (Figure 4B). We next confirmed that METTL7A was significantly downregulated at both the mRNA and protein levels in GC tissues, consistent with our sequencing findings (Figure 4C, D). In most GC cell lines, METTL7A expression was also markedly downregulated (Figure 4E, F). IHC analysis revealed that METTL7A expression was lower in GC tissues than in adjacent noncancerous tissues (Figure 4G). In our in‐house cohort of 93 GC patients, the protein levels of METTL7A and SLC1A5 showed a negative correlation (Figure S5A). Furthermore, METTL7A expression was reduced in GC with more advanced AJCC stage, higher pathological grade, deeper invasion, and lymph node metastasis (Figure S5B–E). Low METTL7A expression was associated with a worse prognosis in GC patients (Figure 4H). The analyses of TCGA and GEO datasets were also consistent with our findings (Figure S5F–H).

To evaluate whether METTL7A regulates SLC1A5 expression, we successfully overexpressed METTL7A in AGS and MKN‐45 GC cells (Figure S5I, J). Western blot, RT‐qPCR, and immunofluorescence staining confirmed that METTL7A markedly suppresses SLC1A5 expression in AGS and MKN‐45 cells (Figure S5K–M). We also examined the effect of METTL7A knockdown on SLC1A5 expression in Jurkat T cells, and no obvious changes were observed (Figure S5N). Subsequently, we aimed to prove that METTL7A attenuates the tumor‐promoting activity of SLC1A5 by suppressing its expression. Colony formation, EdU staining, and CCK‐8 assays demonstrated that the inhibition of GC cell proliferation induced by METTL7A overexpression could be reversed by overexpressing SLC1A5 (Figure 4I, J and Figure S5O). Next, we investigated whether METTL7A overexpression in GC cells could increase glutamine availability in CD8^+^ T cells by downregulating SLC1A5 expression. A co‑culture system was established using MKN‑45 cells and activated human CD8^+^ T cells. Overexpression of METTL7A in MKN‑45 cells led to a decrease in intracellular glutamine levels in GC cells, whereas co‑cultured CD8^+^ T cells exhibited markedly increased intracellular glutamine levels and an increased percentage of Ki67^+^ CD8^+^ T cells (Figure S3F, G). Consequently, the secretion of cytotoxic effectors and cytokines (IFN‑γ, TNF‑α, and GZMB) from CD8^+^ T cells was significantly enhanced, and GC cell survival was suppressed. Notably, these effects were partially reversed by additional overexpression of SLC1A5 (Figure 4K–O and Figure S5P, Q).

Next, we investigated the underlying mechanism by which METTL7A suppresses SLC1A5 expression in GC. The METTL family, particularly the METTL3/METTL14 complex, functions as an m6A writer [28]. The role of METTL7A in cancer has not been well characterized. We first examined whether METTL7A affects global m6A modifications in GC cells. A dot blot assay showed that METTL7A overexpression increased global m6A levels in both AGS and MKN‑45 cells (Figure 4P). Furthermore, METTL7A overexpression enhanced total m6A methyltransferase activity in these cells (Figure S5R). Prior work confirmed that the m6A writer METTL3 modifies SLC1A5 mRNA [21]. To test whether METTL7A acts by upregulating METTL3 expression, we measured METTL3 expression upon METTL7A overexpression and observed no significant changes (Figure S5S, T). MeRIP‐qPCR further confirmed that METTL7A overexpression resulted in elevated m6A enrichment on SLC1A5 mRNA (Figure S5U). RNA stability analysis demonstrated that METTL7A overexpression decreased the stability of SLC1A5 mRNA in GC cells (Figure 4Q). These results suggest that METTL7A overexpression elevates SLC1A5 mRNA m6A modification, thereby promoting its degradation. It has been reported that elevated m6A levels in the 3′ UTR of mRNA can reduce mRNA stability [28]. Next, we designed three pairs of specific primers targeting four very high confidence m6A sites (2300, 2395, 2797, and 2817) in the 3′ UTR of the SLC1A5 mRNA. Two sites (2797 and 2817) were in close proximity and thus assayed by a single primer pair (Figure S5V). The MeRIP‐qPCR results confirmed that METTL7A specifically enhanced m6A modifications at all three regions (covering all four sites) within the 3′ UTR of SLC1A5 mRNA (Figure 4R). To identify the m6A site mediating METTL7A‐dependent SLC1A5 mRNA destabilization, we inserted the 3′ UTR of the SLC1A5 mRNA WT or m6A site mutant (Mut1, Mut2, Mut3, and Mut4) sequence into a luciferase reporter (Figure 4S). The results demonstrated that luciferase activity was reduced when reporter plasmids containing the SLC1A5 3′ UTR‐WT, Mut2, Mut3, or Mut4 sequences were introduced into METTL7A‐overexpressing cells. In contrast, no significant difference in luciferase activity was observed for the reporter containing the SLC1A5 3′ UTR‐Mut1 (Figure 4T). Taken together, our data suggest that METTL7A upregulates m6A modification on the 3′ UTR of SLC1A5 mRNA, destabilizing its mRNA and downregulating its expression. Consequently, this inhibits GC cell proliferation and enhances CD8^+^ T cell antitumor immunity.

### 2.5 METTL7A Suppresses SLC1A5 N‐Glycosylation by Downregulating B4GALT5 in GC Cells

**FIGURE 5:**
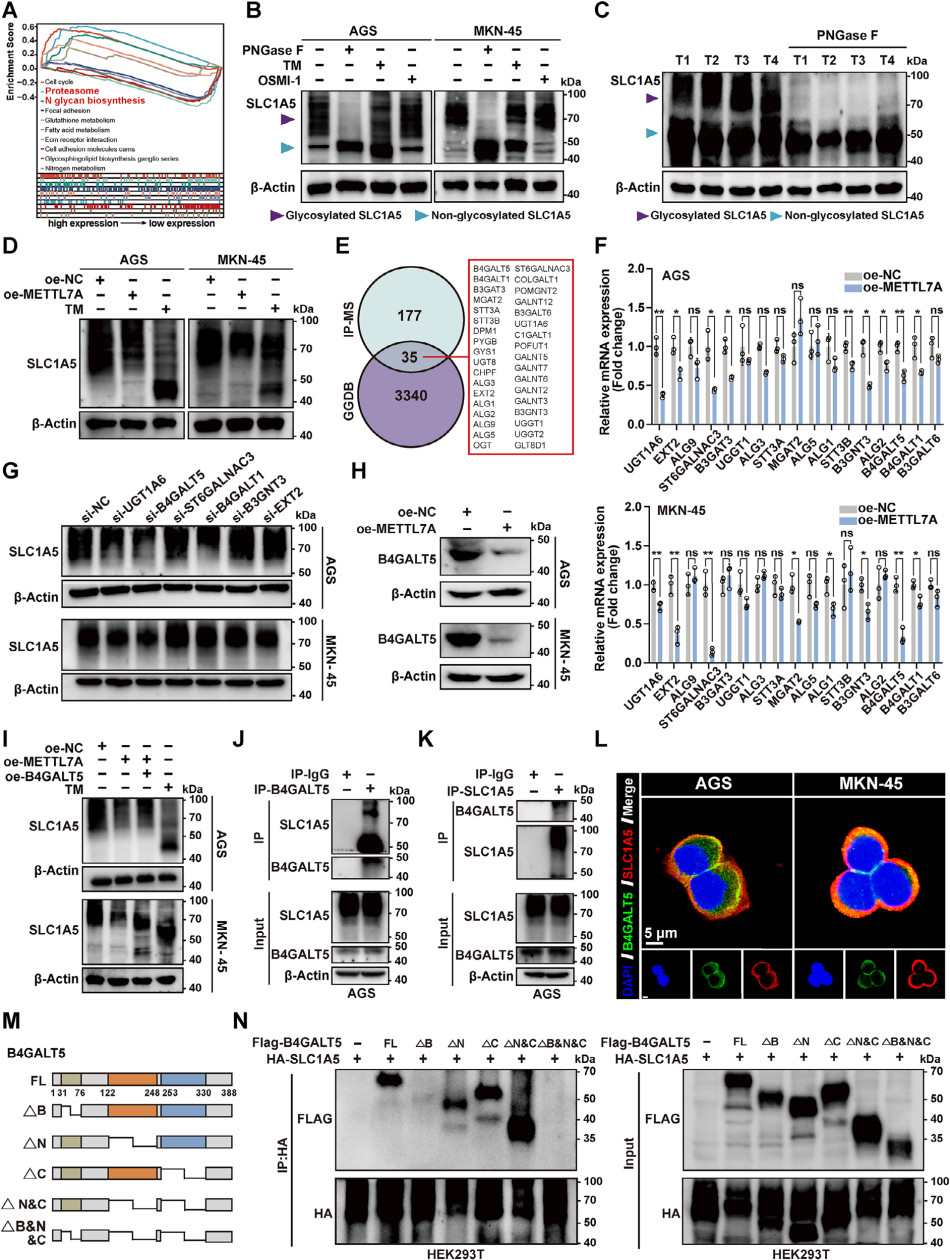
METTL7A inhibits N‐glycosylation of SLC1A5 via downregulation of B4GALT5. (A) GSEA of the GSE84437 dataset based on SLC1A5 expression. (B) Western blot analysis of SLC1A5 glycosylation status in AGS and MKN‐45 cells treated with PNGase F, TM, or OSMI‐1. (C) Western blot analysis of SLC1A5 N‐glycosylation from four human GC tissues treated with or without PNGase F. (D) Western blot analysis of SLC1A5 N‐glycosylation in AGS and MKN‐45 cells with or without METTL7A overexpression, alongside TM treatment. (E) Venn diagram identifying the overlap between SLC1A5‐interacting proteins (by IP‐MS) and glycosyltransferases from the GlycoGene (GGDB) database. (F) qRT‐PCR was used to compare the expression of 17 candidate N‐glycosyltransferases in GC cells with and without METTL7A overexpression. n = 3. (G) Western blot analysis of SLC1A5 N‐glycosylation in AGS and MKN‐45 cells following knockdown of the six indicated N‐glycosyltransferases. (H) Western blot analysis of B4GALT5 protein levels in GC cells with or without METTL7A overexpression. (I) Western blot analysis of SLC1A5 N‐glycosylation under the indicated conditions. (J, K) Co‐immunoprecipitation (Co‐IP) assay probing the interaction between endogenous B4GALT5 and SLC1A5 in AGS cells. (L) Immunofluorescence staining showing the subcellular localization and co‐localization of B4GALT5 and SLC1A5 in AGS and MKN‐45 cells. Scale bars: 5 µm. (M) Schematic diagram of full‐length (FL) and truncated mutants of the B4GALT5 protein. (N) Co‐IP assays assessing the interaction between SLC1A5 and the series of B4GALT5 mutants.

To explore broader functional associations of SLC1A5 in GC, we performed Gene Set Enrichment Analysis (GSEA) on the published GSE84437 dataset after stratifying tumors by SLC1A5 expression. This analysis revealed that SLC1A5 expression was positively correlated with N‑glycan biosynthesis and cell cycle progression, but negatively correlated with proteasome activity (Figure 5A). A previous study has reported that SLC1A5 is a highly N‐glycosylated protein [22]. However, whether SLC1A5 undergoes N‐glycosylation and the functional role of this modification in GC remain unclear. We therefore examined the N‐glycosylation status of SLC1A5 in GC. We found that treatment of AGS and MKN‐45 cell lysates with peptide N‐glycosidase F (PNGase F) or N‐glycosylation inhibitor tunicamycin (TM) completely inhibited endogenous SLC1A5 N‐glycosylation, reducing its relative molecular mass from ∼100–50 kDa to ∼50–40 kDa. In contrast, the O‐GlcNAc transferase inhibitor 1 (OSMI‐1) had no significant effect (Figure 5B). A similar result was observed in four GC tissues. Specifically, PNGase F treatment reduced the molecular weight of SLC1A5 (Figure 5C). Therefore, SLC1A5 undergoes N‐glycosylation in GC.

Interestingly, we found that METTL7A overexpression inhibited SLC1A5 N‐glycosylation in GC cells (Figure 5D). Since glycosyltransferases are the enzymes responsible for protein glycosylation [29], we hypothesized that METTL7A acts by regulating an N‐glycosyltransferase to inhibit SLC1A5 N‐glycosylation. Next, we performed immunoprecipitation coupled with mass spectrometry (IP‐MS) to identify proteins that interact with SLC1A5. Subsequently, the intersection of these results with the GlycoGene (GGDB) database identified 35 glycosyltransferases (17 N‐glycosyltransferases) that may interact with SLC1A5 (Figure 5E). To identify which N‐glycosyltransferase showed expression changes upon METTL7A overexpression and potentially interacted with SLC1A5, we examined the mRNA levels of 17 candidate N‐glycosyltransferases upon METTL7A overexpression. We further discovered that METTL7A overexpression suppressed the mRNA expression of B4GALT5, B4GALT1, UGT1A6, B3GNT3, EXT2, and ST6GALNAC3 (Figure 5F). Next, we individually knocked down these six N‐glycosyltransferases and found that only B4GALT5 knockdown reduced the N‐glycosylation of SLC1A5 in AGS and MKN‐45 cells (Figure 5G and Figure S6A). Correlation analysis revealed a significant positive correlation between B4GALT5 and SLC1A5 in GC (Figure S6B). In addition, METTL7A overexpression suppressed B4GALT5 protein expression (Figure 5H). The inhibitory effect of METTL7A on SLC1A5 N‐glycosylation could be largely rescued by overexpressing B4GALT5 (Figure 5I and Figure S6C, D). Moreover, molecular docking analysis predicted a potential binding interaction between B4GALT5 and SLC1A5 (Figure S6E, F). Co‐immunoprecipitation (Co‐IP) and immunofluorescence staining further confirmed that B4GALT5 physically interacted with SLC1A5 (Figure 5J–L).

To determine which regions of B4GALT5 are critical for its interaction with SLC1A5, we created five truncated B4GALT5 mutants: ΔB (deletion of residues 31–76), ΔN (deletion of residues 122–248), ΔC (deletion of residues 253–330), ΔN&C (deletion of residues 122–248 and 253–330), and ΔB&N&C (deletion of residues 31–76, 122–248, and 253–330) (Figure 5M). The results showed that the B4GALT5 full‐length (FL), ΔN, ΔC, and ΔN&C all physically interacted with SLC1A5. In contrast, ΔB and ΔB&N&C failed to interact with SLC1A5 (Figure 5N). This suggests that B4GALT5 interacts with SLC1A5 through the 31–76 region of B4GALT5. In addition, we confirmed that both mRNA and protein levels of B4GALT5 were significantly upregulated in GC tissues and cell lines (Figure S6G–K). Analyses of the TCGA and GEO databases demonstrated that B4GALT5 was significantly upregulated in GC and was associated with poor patient prognosis (Figure S6L–N). Moreover, activated CD8^+^ T cells expressed much lower METTL7A but higher B4GALT5 than GC cells (Figure S6O). Thus, METTL7A reduces SLC1A5 N‐glycosylation by downregulating B4GALT5.

### 2.6 B4GALT5 Protects SLC1A5 From K48‐Linked Ubiquitination Through N‐Glycosylation at Asparagine 212 in GC Cells

**FIGURE 6:**
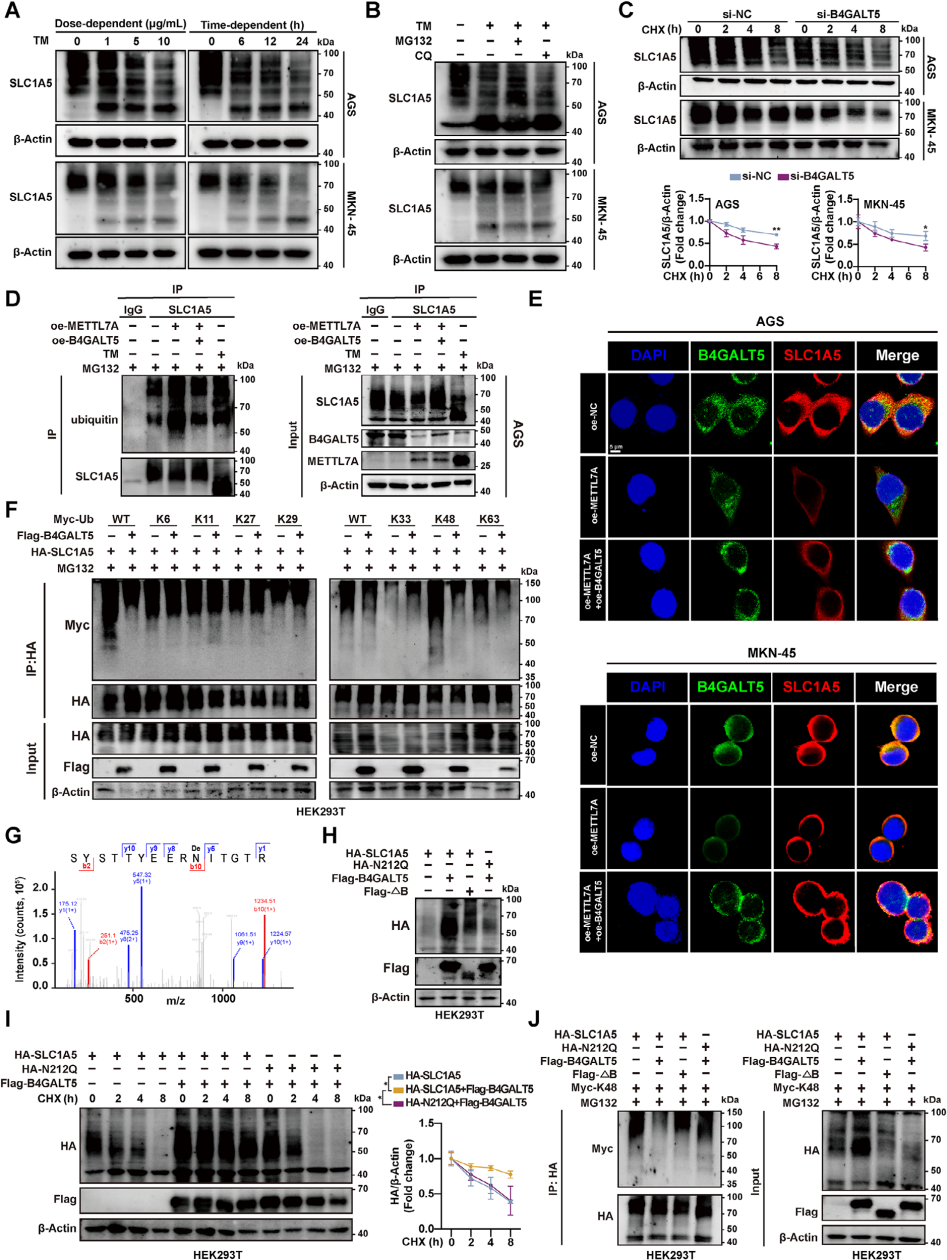
B4GALT5 prevents K48‐linked polyubiquitination of SLC1A5 by promoting its N‐glycosylation. (A) Western blot analysis of SLC1A5 expression in AGS and MKN‐45 cells treated with TM at different doses or for different amounts of time. (B) Western blot analysis of SLC1A5 expression in GC cells treated with TM in combination with the proteasome inhibitor MG132 or the lysosome inhibitor chloroquine (CQ). (C) Cycloheximide (CHX) chase assay assessed by Western blot to determine SLC1A5 protein half‐life in GC cells with or without B4GALT5 knockdown. (D) Evaluation of endogenous SLC1A5 ubiquitination in AGS cells under the indicated conditions. (E) Immunofluorescence staining of SLC1A5 protein levels and localization in AGS and MKN‐45 cells under the indicated conditions. Scale bars: 5 µm. (F) A set of Myc‐tagged ubiquitin mutants (WT, K6, K11, K27, K29, K33, K48, or K63) was co‐expressed with HA‐SLC1A5 and Flag‐B4GALT5 in HEK293T cells, followed by anti‐HA immunoblot to determine the chain linkage pattern on SLC1A5. (G) IP‐MS analysis identified N‐glycosylation at the N212 site of SLC1A5. (H) Western blot analysis of SLC1A5 N‐glycosylation in HEK293T cells co‐transfected with WT or N212Q mutant SLC1A5 and either full‐length (FL) or truncated (ΔB) B4GALT5. (I) CHX chase assay assessed by Western blot comparing the degradation rate of WT and N212Q mutant SLC1A5 protein with or without B4GALT5 overexpression. (J) HEK293T cells were co‐transfected with HA‐SLC1A5 (WT or N212Q), Flag‐B4GALT5 (FL or ΔB mutant), and Myc‐K48 ubiquitin. Immunoprecipitation of HA‐tagged SLC1A5 to analyze its K48‐linked polyubiquitination.

The role of N‐glycosylation in regulating SLC1A5 function was then investigated. Protein N‐glycosylation can modulate protein stability and receptor‐ligand interactions [30]. Our initial GSEA revealed significant enrichment of the proteasome pathway in the SLC1A5 low‐expression group (Figure 5A). Based on this and the established role of N‐glycosylation in degradation, we proposed that N‐glycosylation protected SLC1A5 from ubiquitin‐proteasome degradation. First, in both AGS and MKN‐45 cells, the hyperglycosylated form and total protein levels of SLC1A5 were reduced in a TM dose‐ and time‐dependent manner (Figure 6A). The TM‐induced downregulation of SLC1A5 protein was reversed by the proteasome inhibitor MG132 but not by the lysosome inhibitor chloroquine (CQ) (Figure 6B). The above results indicate that N‐glycosylation of SLC1A5 protects it from proteasome‐dependent degradation. We then assessed the effect of B4GALT5 or METTL7A expression on endogenous SLC1A5 stability using a protein synthesis inhibitor cycloheximide (CHX) chase assay. It was shown that B4GALT5 knockdown or METTL7A overexpression significantly reduced SLC1A5 protein stability (Figure 6C and Figure S7A). Furthermore, METTL7A overexpression or TM treatment elevated SLC1A5 ubiquitination, and this METTL7A‐induced ubiquitination was reversed by B4GALT5 co‐overexpression (Figure 6D). Immunofluorescence staining also revealed that METTL7A overexpression reduced SLC1A5 protein levels, whereas co‐overexpression of B4GALT5 largely restored its expression (Figure 6E). These results suggest that METTL7A suppresses B4GALT5‐mediated SLC1A5 N‐glycosylation, thereby promoting SLC1A5 ubiquitination and proteasome‐dependent degradation. We next identified the specific ubiquitin chain linkage on SLC1A5 regulated by B4GALT5. Our data confirm that B4GALT5 specifically enhances K48‐linked polyubiquitination of SLC1A5, but not other chain types (Figure 6F). K48‐linked ubiquitination is known to serve as the canonical signal for proteasomal degradation [31], which is consistent with our earlier data. These results indicate that METTL7A impairs SLC1A5 N‐glycosylation via B4GALT5, thereby promoting SLC1A5 K48‐linked ubiquitination and proteasomal degradation.

To identify the critical asparagine (Asn) residue on SLC1A5 targeted by B4GALT5 for N‐glycosylation, our IP‐MS analysis revealed Asn212 as the unique differentially N‐glycosylated residue of SLC1A5 (Figure 6G). To test the importance of this Asn site, we further constructed a single Asn212 site mutant in which the asparagine (N) residue was changed to glutamine (Q), designated N212Q. The N212Q mutation was found to decrease B4GALT5‐mediated SLC1A5 N‐glycosylation. Furthermore, the B4GALT5 ΔB (deletion of residues 31–76) mutant similarly reduced this modification (Figure 6H), demonstrating that the 31–76 region of B4GALT5 mediates binding to SLC1A5 to catalyze N‐glycosylation at Asn212. Notably, we identified an additional potential N‑glycosylation site at Asn163 in SLC1A5 using the UniProt database. To determine whether this site also contributes to B4GALT5‑mediated modification, we constructed an N163Q mutant. However, this mutation did not impair B4GALT5‑dependent N‑glycosylation of SLC1A5, indicating that Asn163 is not required for this modification (Figure S7B). To confirm that B4GALT5 increases SLC1A5 N‐glycosylation at Asn212, thereby preventing SLC1A5 ubiquitin‐proteasome degradation, we performed a CHX‐chase assay. The results revealed that B4GALT5 prolonged the half‐life of SLC1A5 protein, whereas the N212Q mutation accelerated SLC1A5 protein degradation (Figure 6I). Next, we co‐expressed HA‐SLC1A5, FL or ΔB mutant of Flag‐B4GALT5, and Myc‐K48‐linked ubiquitin (Myc‐K48) in HEK293T cells. Flag‐B4GALT5 expression markedly decreased the K48‐linked ubiquitination of HA‐SLC1A5. In contrast, the ΔB mutant significantly enhanced HA‐SLC1A5 K48‐linked ubiquitination. Moreover, the N212Q mutation significantly increased SLC1A5 K48‐linked ubiquitination, largely attenuating the stabilizing effect of B4GALT5 (Figure 6J). In conclusion, B4GALT5 enhances SLC1A5 N‐glycosylation at Asn212, leading to the suppression of K48‐linked ubiquitination and subsequent accumulation of SLC1A5 protein.

### 2.7 Luteolin Suppresses GC Progression by Upregulating METTL7A and Enhances the Efficacy of Anti‐PD‐1 Therapy

**FIGURE 7:**
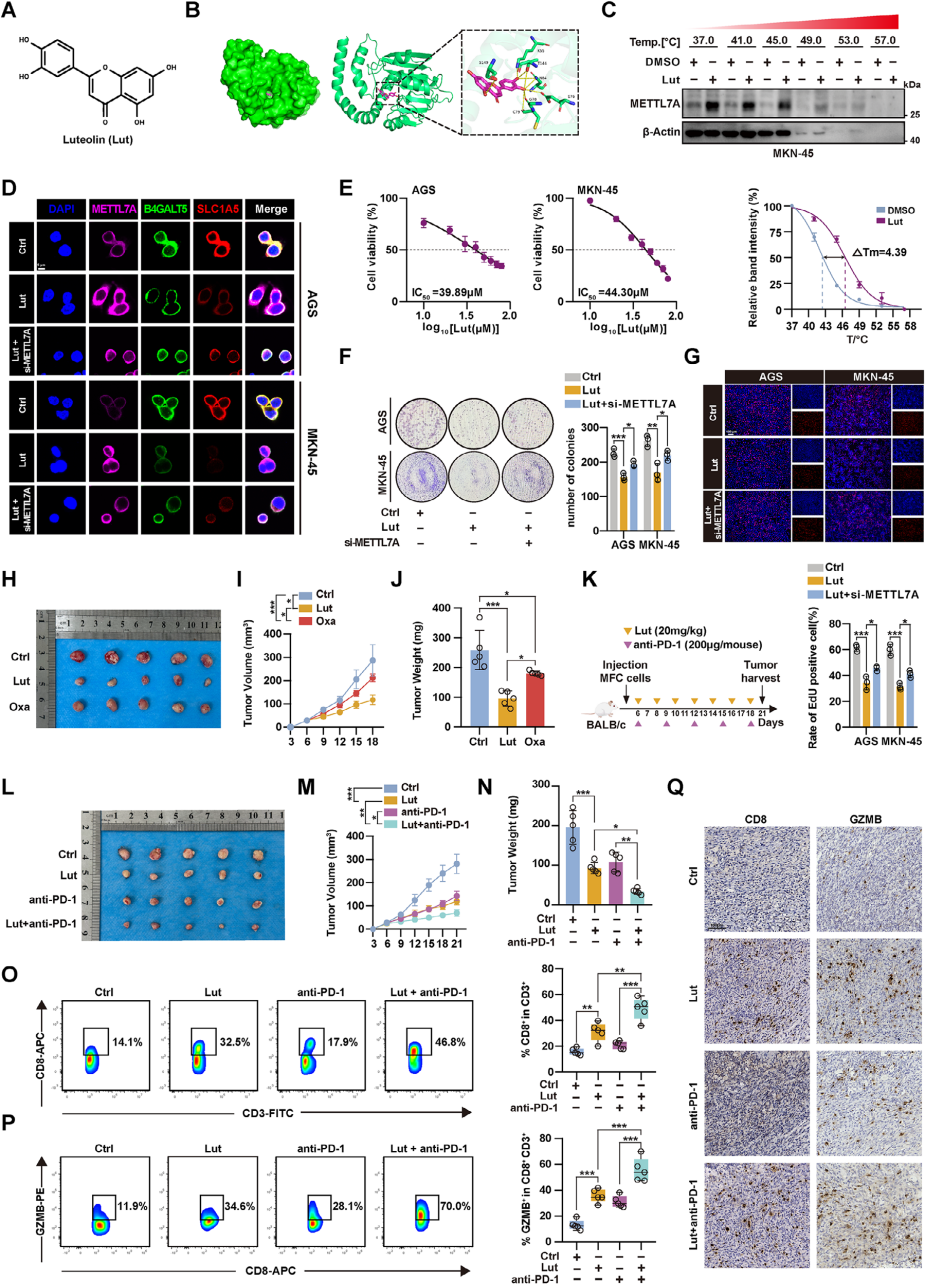
Luteolin inhibits GC progression by upregulating METTL7A and enhances the efficacy of anti‐PD‐1 therapy. (A) Chemical structure of the natural flavonoid Luteolin (Lut). (B) Molecular docking model predicting the binding pose of Lut within the METTL7A protein structure. (C) Cellular Thermal Shift Assay (CETSA) measuring the thermal stability of METTL7A protein in GC cells treated with DMSO or Lut. (D) Immunofluorescence staining of METTL7A, B4GALT5, and SLC1A5 protein levels in AGS and MKN‐45 cells under the indicated conditions. Scale bars: 5 µm. (E) The half‐maximal inhibitory concentration (IC_50_) values of Lut in AGS and MKN‐45 cells. n = 3. (F, G) Colony formation (F) and EdU staining (G) assays in AGS and MKN‐45 cells under the indicated conditions. Scale bars: 100 µm. n = 3. (H–J) Representative images (H), tumor growth curves (I), and tumor weights (J) in MFC xenograft‐bearing mice across different treatment groups. n = 5. (K) Schematic of the combination therapy experiment timeline using MFC tumor‐bearing mice treated with Lut alone, anti‐PD‐1 antibody alone, or their combination. (L–N) Representative images (L), tumor growth curves (M), and tumor weights (N) in MFC xenograft‐bearing mice across different treatment groups. n = 5. (O, P) Flow cytometry analysis of tumor‐infiltrating CD8^+^ T cells (O) and GZMB^+^ CD8^+^ T cells (P) across different treatment groups. n = 5. (Q) Representative IHC images showing CD8 and GZMB staining across different treatment groups. Scale bars: 100 µm.

To develop a novel therapy targeting the METTL7A/SLC1A5 axis, we investigated agents that could upregulate METTL7A. Building on our team's previous work with antitumor flavonoids in GC, we focused on natural flavonoid luteolin (Lut) and investigated whether it suppresses GC progression by targeting METTL7A (Figure 7A) [32, 33, 34]. Molecular docking analysis predicted a potential binding association between Lut and METTL7A (affinity: –6.48 kcal/mol). Lut was predicted to form multiple hydrogen bonds with key residues of METTL7A (Figure 7B and Figure S8A). To examine whether Lut engages METTL7A in GC cells, we performed a Cellular Thermal Shift Assay (CETSA). The results demonstrated that Lut treatment caused a significant rightward shift in the thermal denaturation curve, indicating that Lut substantially enhanced the thermal stability of METTL7A (ΔTm = 4.39°C) (Figure 7C). Furthermore, Lut increased METTL7A expression, with a consequent reduction in B4GALT5 and SLC1A5 expression in GC cells, while METTL7A knockdown in Lut‐treated GC cells largely reversed the suppressive effect on the expression of B4GALT5 and SLC1A5 (Figure 7D and Figure S8B–E). To determine whether Lut relieves glutamine competition via SLC1A5 downregulation, we pretreated MKN‑45 cells with Lut and then co‑cultured them with activated CD8^+^ T cells. Lut pretreatment decreased intracellular glutamine levels in the GC cells, while co‑cultured CD8^+^ T cells exhibited elevated intracellular glutamine and restored proliferative capacity (Figure S3F, G). Moreover, Lut significantly inhibited GC cell proliferation in vitro, and this inhibitory effect was reversed by METTL7A knockdown (Figure 7F, G and Figure S8F, G).

We next examined the antitumor effects of Lut in GC models in vivo. Lut significantly suppressed tumor growth in vivo, in a dose‐dependent manner (Figure S8H–J). Critically, Lut treatment induced no observable histopathological changes or signs of toxicity in the examined major organs (heart, lungs, liver, spleen, and kidneys) (Figure S8K). To evaluate the clinical potential of Lut, we compared its efficacy with the first‐line chemotherapeutic agent oxaliplatin (Oxa) in the subcutaneous xenograft model. The results demonstrated that Lut (20 mg/kg) induced a greater inhibition of tumor growth than Oxa (5 mg/kg) (Figure 7H–J). Moreover, in the subcutaneous tumors, Lut treatment increased METTL7A protein expression and decreased SLC1A5 and B4GALT5 protein expression (Figure S8L). METTL7A overexpression significantly suppressed subcutaneous tumor progression (Figure S8M–Q). These results reveal that Lut exerts tumor‐suppressive effects in GC at least in part by upregulating METTL7A, which acts as a tumor suppressor to repress the key oncogene SLC1A5.

In advanced GC, anti‐PD‐1 therapy improves patient survival, and this efficacy is positively correlated with the functional status of CD8^+^ T cells [3, 35]. Given that Lut increases glutamine availability in CD8^+^ T cells, we examined whether Lut enhances the efficacy of anti‑PD‑1 therapy. To test this hypothesis, we first examined PD‑L1 expression in Lut‑treated GC cells and found no significant change (Figure S8R). Subsequently, we established subcutaneous xenograft mouse models using MFC cells, which were then treated with Lut alone, anti‐PD‐1 antibody alone, or a combination of both (Figure 7K). Compared with either monotherapy, the combinational treatment group showed significantly reduced tumor volume and weight (Figure 7L–N). Moreover, this group markedly promoted intratumoral infiltration of both total CD8^+^ T cells and GZMB^+^ CD8^+^ T cells, with corresponding increases in serum levels of cytotoxic effectors and cytokines (Figure 7O–Q and Figure S8S, T). These findings suggest that combining Lut with anti‐PD‐1 therapy offers a more effective therapeutic strategy for GC treatment.

## 3 Discussion

To discover critical factors driving GC progression, transcriptomic and proteomic analyses of paired GC and adjacent noncancerous tissues were performed. We then identified SLC1A5 as a significantly upregulated gene in GC. Tumor cells rewire metabolism to meet proliferative demands under nutrient limitation [36]. The central role of glutamine metabolism in tumor progression frequently leads to a profound tumor dependency, termed “glutamine addiction” [25, 26, 37]. Since SLC1A5 serves as the primary cellular transporter for glutamine [38], we hypothesized that its overexpression supports the heightened glutamine demand of GC cells. Consistent with this hypothesis, our results demonstrate that SLC1A5 promotes GC cell proliferation by enhancing glutamine accumulation.

Immunotherapy has achieved significant advances in oncology. However, it only benefits a subset of GC patients, leaving most patients with “immune cold” tumors that are refractory to current immunotherapies [1, 39]. Therefore, converting TME from an “immune cold” state into an “immune hot” state represents a promising strategy. Accumulating evidence indicates that aberrant glutamine metabolism is a key driver of the “immune cold” TME phenotype [40, 41, 42, 43]. Intriguingly, our in vivo studies revealed that glutamine plays a dual role in tumor progression. Glutamine supplementation promoted tumor growth in immunodeficient mice but suppressed it in immunocompetent mice. This stark contrast indicates that the antitumor effect of glutamine was at least partially mediated by the immune system. Importantly, glutamine supplementation significantly enhanced intratumoral infiltration and function of CD8^+^ T cells. These findings suggest that glutamine supplementation restrains tumor progression by enhancing CD8^+^ T cell function. Given that competition for nutrients within the TME can severely compromise immune cell function [12, 13, 27, 44], we hypothesized that GC cells may outcompete CD8^+^ T cells for glutamine. Indeed, co‐culturing GC cells with CD8^+^ T cells significantly reduced glutamine levels and impaired proliferative capacity in the CD8^+^ T cells. Moreover, CD8^+^ T cell function was markedly impaired following exposure to the GC cell‐conditioned medium, and this effect was rescued by glutamine supplementation. We next sought to define whether this glutamine competition relies on SLC1A5. Notably, SLC1A5 knockdown reduced intracellular glutamine levels in GC cells, while restoring glutamine abundance and enhancing effector function in co‑cultured CD8^+^ T cells. Furthermore, upon SLC1A5 knockdown, glutamine supplementation no longer exerted additional effects on tumor progression or CD8^+^ T cell function, supporting SLC1A5 as a critical mediator of glutamine competition in GC. This was further corroborated by our in vivo data. Although activated CD8^+^ T cells upregulate SLC1A5 and depend on glutamine metabolism [45], our data showed that GC cells express higher SLC1A5 levels than activated CD8^+^ T cells, and this advantage would be further magnified in the TME, where TCR signals are considerably weaker. Moreover, published research has revealed that tumor cells actively stabilize SLC1A5 protein under glutamine‐limited stress [46], potentially reinforcing their competitive edge in the TME. Functionally, glutamine deprivation has been shown to impair T cell activation [47], whereas our co‐culture data further demonstrate that SLC1A5‐mediated glutamine competition suppresses CD8^+^ T cell proliferation and effector function. Thus, SLC1A5‐driven glutamine competition potently suppresses CD8^+^ T cell proliferation and effector function while potentially compromising their activation in the TME. While our glutamine quantification data establish a functional link between SLC1A5 and glutamine competition, these endpoint measurements reflect intracellular abundance and cannot distinguish uptake from utilization or efflux. Nonetheless, the reduced CD8^+^ T cell glutamine levels and concurrent functional suppression observed in our data point toward restricted external glutamine supply as an important contributing factor. ^1^ ^3^C‑glutamine tracing represents a valuable complementary approach to dissect the metabolic flux controlled by this axis. Additionally, exploration of other glutamine transporters (such as SLC38A1) and SLC1A5 mutants would further clarify SLC1A5's transport specificity.

Our findings demonstrate that SLC1A5 plays dual roles in GC, orchestrating GC cell metabolism and fostering an immunosuppressive microenvironment. Hence, elucidating the mechanisms underlying SLC1A5 overexpression represents a critical research objective. Emerging evidence indicates that m6A modification of SLC1A5 mRNA exhibits disease‐specific patterns. Despite these findings, the upstream regulatory mechanisms controlling SLC1A5 expression in GC are poorly understood. For example, in acute myeloid leukemia, pancreatic cancer, and osteoporosis, IGF2BP2 binds to and further stabilizes m6A‐modified SLC1A5 mRNA [18, 19, 21]. Conversely, in clear cell renal cell carcinoma, removal of m6A modification by FTO results in increased SLC1A5 expression [20]. To date, only STAT3 signaling and the circAKT3/miR‐515‐5p axis have been reported to regulate SLC1A5 expression in GC [15, 48], underscoring the limited knowledge in this regard. In this study, we verified m6A modification on SLC1A5 mRNA in GC. To identify potential m6A regulators of SLC1A5, we performed an integrated screening of the m6A2Target database and our sequencing data, which revealed METTL7A as the sole candidate. Notably, METTL7A is a member of the METTL family, and its m6A‐related role remains uncharacterized in GC. We demonstrated that METTL7A was significantly downregulated in GC. METTL7A overexpression increased global m6A methyltransferase activity and total m6A levels in GC cells, and destabilized SLC1A5 mRNA by increasing m6A modifications within its 3′ UTR. However, whether METTL7A functions as a canonical m6A methyltransferase in GC remains to be determined. Additionally, increased m6A deposition on SLC1A5 mRNA is correlated to METTL7A upregulation, but whether METTL7A directly or indirectly methylates SLC1A5 mRNA remains elusive. Functionally, reduced METTL7A expression increased intracellular glutamine levels in GC cells, while decreasing glutamine abundance in co‑cultured CD8^+^ T cells, thereby promoting GC cell proliferation and impairing CD8^+^ T cell function via upregulating SLC1A5 expression.

N‐glycosylation modulates protein folding, stability, and localization [30]. Aberrant N‐glycosylation is a hallmark of cancer and contributes to malignant phenotypes such as metabolic reprogramming, immune evasion, and ferroptosis resistance [49, 50, 51]. Although SLC1A5 is a highly N‐glycosylated protein, its glycosylation has been implicated primarily in HIV‐1 gp160 complex assembly [52], and its role in tumor progression remains elusive. In this study, we identified specific N‐glycosylation of SLC1A5 in GC. Notably, METTL7A significantly suppressed SLC1A5 N‐glycosylation, suggesting that it may act through modulating specific N‐glycosyltransferases. We therefore screened candidate enzymes and identified B4GALT5 as the critical N‐glycosyltransferase responsible for SLC1A5 modification. B4GALT5 expression decreased upon METTL7A overexpression, and B4GALT5 directly mediated the N‐glycosylation of SLC1A5. Nevertheless, the precise mechanism by which METTL7A decreased B4GALT5 warrants further investigation.

Tumor cells frequently utilize N‐glycosylation to stabilize oncoproteins, with PD‐L1 and EREG being prominent examples [53, 54]. However, the role of SLC1A5 N‐glycosylation in GC has not been fully elucidated. We found that SLC1A5 expression levels were positively correlated with its high N‐glycosylation. Protein turnover in cells is primarily mediated through proteasomal and lysosomal degradation [55]. We showed that tunicamycin (TM)‐induced degradation of SLC1A5 was specifically rescued by the proteasome inhibitor MG132, but not by the lysosome inhibitor chloroquine, indicating that N‐glycosylation protects SLC1A5 from proteasomal degradation. Functionally, METTL7A decreases B4GALT5 expression, leading to reduced N‐glycosylation at the N212 site, which in turn promotes K48‐linked polyubiquitination and subsequent proteasomal degradation of SLC1A5. We also found that METTL7A suppressed SLC1A5 expression more potently than TM, suggesting that METTL7A acts through both N‑glycosylation‑related and m6A‑dependent pathways to regulate SLC1A5. Collectively, restoring METTL7A expression may inhibit SLC1A5, representing a therapeutic strategy to simultaneously target the dual oncogenic role of SLC1A5 in fueling tumor metabolism and enforcing immune evasion.

Targeting glutamine metabolism has emerged as a crucial strategy in antitumor drug development, prompting the exploration of agents such as the GLS1 inhibitor CB‐839 and the SLC1A5 inhibitor V‐9302 [24]. Currently, the next‐generation inhibitor V‐9302 faces potential limitations in efficacy and a risk of resistance [56]. Therefore, targeting glutamine transport alone may be insufficient, and strategies aiming to suppress SLC1A5 overexpression may offer greater therapeutic benefit in GC. Natural flavonoids, which exhibit antitumor activity and favorable safety profiles, represent promising candidates for clinical development [33, 57]. Luteolin (Lut), a natural flavonoid, has demonstrated antitumor effects in various malignancies including esophageal cancer and glioblastoma [33, 34, 58]. We found that Lut significantly inhibited GC progression without inducing apparent systemic toxicity. Mechanistically, Lut promoted METTL7A expression, leading to SLC1A5 downregulation and suppression of GC progression. Furthermore, Lut enhanced intratumoral infiltration of cytotoxic T cells and potentiated the efficacy of anti‑PD‑1 therapy. The prior literature has confirmed direct immunomodulatory effects of Lut on CD8^+^ T cells [59]. Our data demonstrated that Lut indirectly enhanced CD8^+^ T cell function through the METTL7A/SLC1A5 axis in GC cells by alleviating glutamine competition. These findings suggest that Lut may modulate CD8^+^ T cells through both indirect and direct mechanisms. Future investigation of Lut's direct effects on CD8^+^ T cell biology and the potential role of the METTL7A/SLC1A5 axis in CD8^+^ T cells will further clarify its mechanisms and clinical translation. Validation in orthotopic models, genetically engineered mice, or humanized immune systems would further strengthen translational relevance.

**FIGURE 8:**
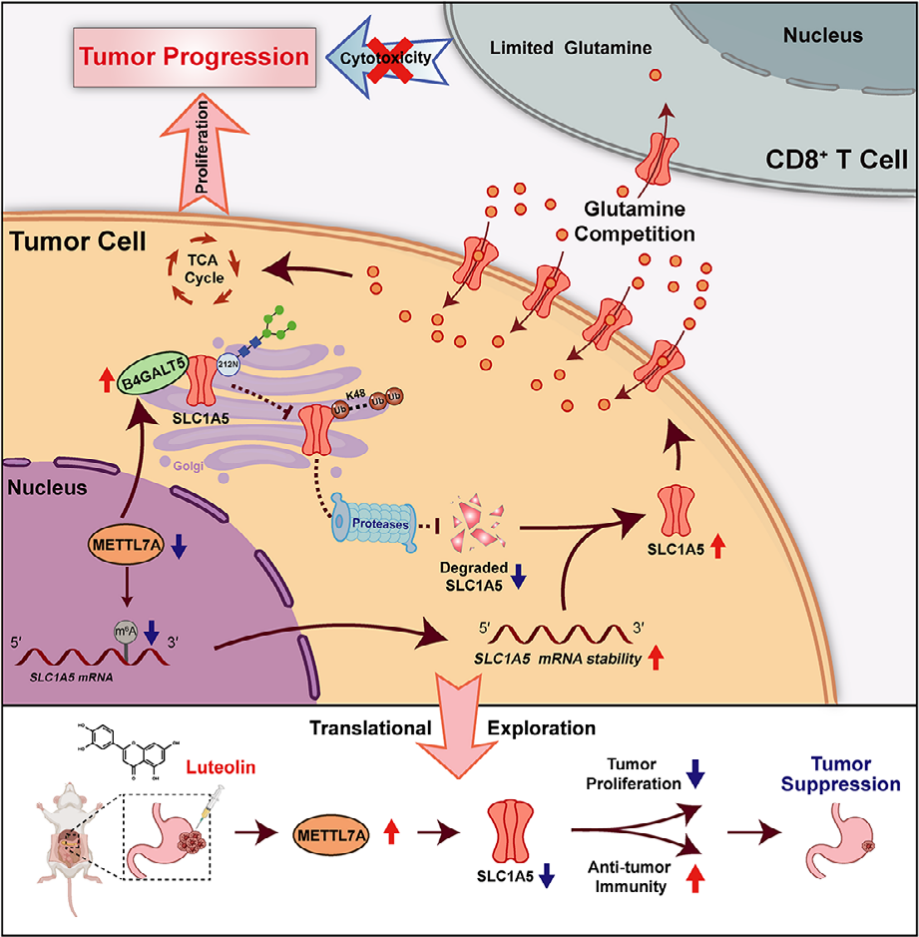
Schematic diagram of this study.

Overall, GC cells enhance glutamine accumulation by upregulating SLC1A5 expression. This upregulation not only directly promotes GC cell proliferation, but also competitively depletes glutamine within the TME, impairing antitumor immunity of CD8^+^ T cells. Mechanistically, reduced METTL7A expression in GC leads to aberrant SLC1A5 overexpression through an m6A‐dependent regulatory mechanism and antagonistic N‐glycosylation–ubiquitination axis. Furthermore, targeting the METTL7A/SLC1A5 axis with the natural flavonoid Lut significantly suppresses GC progression and enhances the efficacy of anti‐PD‐1 therapy (Figure 8). Collectively, these findings establish the METTL7A/SLC1A5 axis as a pivotal regulatory hub linking tumor metabolism and immune evasion in GC, providing a strong mechanistic rationale for the development of precision therapeutic strategies.

GC progression is driven by SLC1A5‐mediated glutamine accumulation, which promotes both tumor cell proliferation and CD8^+^ T cell dysfunction through glutamine competition. This study identifies that low METTL7A drives SLC1A5 overexpression through dual mechanisms. Loss of METTL7A stabilizes SLC1A5 mRNA by reducing its m6A modification. Concurrently, METTL7A deficiency increased B4GALT5 expression. B4GALT5 stabilizes SLC1A5 via N‐glycosylation at the N212 site, which blocks K48‐linked polyubiquitination and proteasomal degradation. Luteolin targets the METTL7A/SLC1A5 axis to inhibit GC progression and enhance the efficacy of anti‐PD‐1 therapy.

## 4 Experimental Section

### 4.1 Cell Lines and Cell Culture

Human GC cell lines (AGS, RRID: CVCL_0139; MKN‐45, RRID: CVCL_0434; HGC‐27, RRID: CVCL_1279; SNU‐1, RRID: CVCL_0099; NCI‐N87, RRID: CVCL_1603), gastric mucosa epithelial cell line (GES‐1, RRID: CVCL_EQ22), mouse forestomach carcinoma cell line (MFC, RRID: CVCL_5J48), Jurkat T cell line (RRID: CVCL_0367), and human embryonic kidney cell line (HEK293T, RRID: CVCL_0063) were from American Type Culture Collection (ATCC). AGS cells were cultured in Ham's F‐12 medium (Gibco) supplemented with 10% fetal bovine serum (FBS; Gibco). HEK293T cells were cultured in DMEM medium (Gibco) supplemented with 10% FBS. Other cell lines were maintained in RPMI‐1640 medium (Gibco) supplemented with 10% FBS. All cells were maintained under standard culture conditions (37°C, 5% CO_2_, humidified atmosphere).

### 4.2 Patients and Specimens

Tumor samples and adjacent nontumor tissues from GC patients and fresh blood samples from healthy donors were obtained at the Fourth Affiliated Hospital of Harbin Medical University. Informed consent was obtained from all participants, including both GC patients and healthy donors (YXLLSC‐2018‐01) (2025‐Ethics Review‐120).

### 4.3 Animal Experiments

All animal experiments were performed in accordance with a protocol approved by the Institutional Animal Care and Use Committee of the Fourth Affiliated Hospital of Harbin Medical University (2025‐DWSYLLCZ‐100). Female BALB/c and BALB/c‐nu/nu mice (4–5 weeks old) were purchased from the Jiangsu Huachuang Xinnuo Pharmaceutical Technology Co., Ltd. For in vivo experiments, MFC cells (1 × 10^6^ cells suspended in 100 µL of PBS) were subcutaneously inoculated into the posterior flanks of mice. Glutamine (MedChemExpress, HY‐N0390R) was given daily via intratumoral injection at a dose of 200 mg/kg. Luteolin (Lut; 10 or 20 mg/kg; MedChemExpress, HY‐N0162) or oxaliplatin (Oxa; 5 mg/kg; MedChemExpress, HY‐17371) was given intraperitoneally every two days. anti‐PD‐1 (BioXcell, BE0146) was given intraperitoneally at 200 µg per mouse every three days. Tumor volumes were measured along the maximum axis (L) and the right‐angle diameter to that axis (W) and were calculated as follows: tumor volume = (L × W^2^)/2.

### 4.4 Human CD8^+^ T Cell Separation, Activation, and Co‐Culture

Human peripheral CD8^+^ T cells were isolated from the peripheral blood mononuclear cells (PBMCs) using the EasySep Human CD8^+^ T Cell Isolation Kit (Stemcell, 17953). Freshly purified human cells were activated with Human CD3/CD28 T Cell Activator (Stemcell, 10971) and cultured in ImmunoCult‐XF T Cell Expansion Medium (Stemcell, 10981) supplemented with IL‐2 (10 ng/mL; Stemcell, 78036) or RPMI‐1640 supplemented with 10% FBS, HEPES (10 mM; MedChemExpress, HY‐D0857), glutamine (2 mM), sodium pyruvate (1 mM; MedChemExpress, HY‐W015913), IL‐2 (10 ng/mL), and 2‐mercaptoethanol (50 µM; MedChemExpress, HY‐Y0326). For co‑culture experiments, MKN‑45 cells and human CD8^+^ T cells were counted using a hemocytometer prior to seeding to ensure accurate cell numbers. Specifically, 0.5 × 10^6^ CD8^+^ T cells and 1 × 10^5^ MKN‑45 cells (ratio = 5:1) were seeded in the upper and lower chambers of a 0.4 µm Transwell system, respectively, and co‑cultured for 48 h. This ratio was chosen based on a previous study [13]. For experiments involving METTL7A overexpression or SLC1A5 knockdown, stable GC cell lines were established prior to co‑culture. For luteolin treatment, GC cells were pretreated with luteolin (40 µM) for 24 h, washed twice with PBS, and then co‑cultured with CD8^+^ T cells as described above. Following stimulation with a cytokine transport inhibitor, CD8^+^ T cells were analyzed by flow cytometry. The viability of MKN‑45 cells was assessed using Annexin V/PI (MedChemExpress, HY‐K1073), Calcein‑AM/PI (Beyotime Biotechnology, C2015S), LDH release (Nanjing Jiancheng, A020‐2‐2), and crystal violet staining (Beyotime Biotechnology, C0121).

### 4.5 Lentiviral and Plasmid Transfection

To establish stable knockdown cells, shRNAs targeting human or mouse SLC1A5 (sequences in Table S1) were cloned into the hU6‐MCS‐CMV‐Puromycin lentiviral vector (GeneChem). To achieve stable overexpression, the coding sequences of B4GALT5 and METTL7A (human and mouse) were chemically synthesized by GeneChem and GenePharma, respectively. Cells were infected with the lentivirus and selected with puromycin (5 µg/mL; MedChemExpress, HY‐B1743) for a week. For transient overexpression, the SLC1A5 cDNA was subcloned into the pcDNA3.1(+) plasmid (GenePharma). For transient knockdown, siRNAs targeting METTL7A, UGT1A6, B4GALT5, B4GALT1, B3GNT3, ST6GALNAC3, or EXT2 (sequences in Table S2) were chemically synthesized (GenePharma).

### 4.6 RNA Sequencing

Total RNA was extracted using TRIzol reagent (Invitrogen, 15596018CN). RNA integrity was confirmed (RIN > 7.0) using a Qubit 3.0 Fluorometer and an Agilent 5300 Fragment Analyzer. Strand‐specific cDNA libraries were prepared following mRNA purification, fragmentation, reverse transcription, second‐strand synthesis using dUTP, adapter ligation, and size selection (average insert size: 400 ± 50 bp). Libraries were PCR‐amplified and subjected to paired‐end sequencing (2 × 150 bp) on an Illumina NovaSeq 6000 platform. Raw read counts were used for differential expression analysis with the DESeq2 package and the edgeR package. Genes with an adjusted *p*‐value (false discovery rate, FDR) < 0.05 and |log_2_Fold Change| ≥ 1 were defined as differentially expressed genes (DEGs).

### 4.7 DIA Proteomics

Total proteins were extracted from tissue samples using urea lysis buffer, followed by reduction, alkylation, and tryptic digestion. The resulting peptides were fractionated by high‐pH reversed‐phase HPLC and analyzed by nano‐liquid chromatography coupled to tandem mass spectrometry (LC‐MS/MS) on a Thermo Astral platform operating in data‐independent acquisition (DIA) mode. Raw DIA data were processed and quantified using the DIA‐NN software against the UniProt database. Genes with an adjusted *p*‐value (false discovery rate, FDR) < 0.05 and |log_2_Fold Change| ≥ 1 were defined as differentially expressed proteins (DEPs).

### 4.8 LC‐MS/MS

To identify SLC1A5‐interacting proteins, Co‐immunoprecipitation (Co‐IP) was performed using an anti‐SLC1A5 antibody in AGS cell lysates. The immunoprecipitated complexes on beads were subjected to on‐bead tryptic digestion. The resulting peptides were analyzed by LC‐MS/MS on a Q Exactive HF system. Raw data were processed using MaxQuant against the UniProt database with 1% FDR filtering. To map the N‐glycosylation sites, SLC1A5 was immunoprecipitated from AGS cell lysates. The immunoprecipitated protein was resolved by SDS‐PAGE, and the target band was excised for in‐gel digestion. The enriched SLC1A5 was digested with trypsin, and the resulting glycopeptides were selectively enriched using affinity chromatography. Enriched glycopeptides were treated with PNGase F (Beyotime Biotechnology, P2318) to remove N‐glycans, converting modified asparagine residues for site mapping. The deglycosylated peptides were analyzed by LC‐MS/MS on a timsTOF HT system operating in data‐independent acquisition (dia‐PASEF) mode. The raw data were searched against the human proteome database using Spectronaut (v18), with N‐glycosylation set as a variable modification. Identifications were filtered at a 1% FDR at the peptide and protein levels.

### 4.9 qRT‐PCR

Total RNA was extracted using TRIzol reagent and reverse‐transcribed into cDNA with the PrimeScript RT Reagent Kit (Takara, RR037A). qRT‐PCR was performed using FastStart Universal SYBR Green Master Mix (Roche, 4913914001) on a real‐time PCR system. β‐actin was used as an endogenous control for normalization. The relative expression of target genes was calculated using the 2^−ΔΔCt^ method. The sequences of the primers are listed in Table S3.

### 4.10 Western Blot

Total protein was extracted from cells or tissues using RIPA lysis buffer (Beyotime Biotechnology, P0013B) supplemented with protease (Beyotime Biotechnology, P1005) and phosphatase (Beyotime Biotechnology, P1081) inhibitor cocktails. Protein concentration was determined using a BCA assay kit (Beyotime Biotechnology, P0012). Equal amounts of protein were separated by SDS‐PAGE and transferred onto PVDF membranes. After blocking, the membranes were incubated overnight at 4°C with specific primary antibodies. The next day, membranes were washed and incubated with HRP‐conjugated secondary antibodies (Absin, abs20040, abs20039; Proteintech, SA00001‐7L) for 1 h at room temperature. Protein bands were visualized using ECL substrate (Meilunbio, MA0187) on a ChemiDoc MP imaging system (Bio‐Rad) and quantified by densitometry with ImageJ software. The following antibodies were used: SLC1A5 (Cell Signaling Technology, 8057; Proteintech, 20350‐1‐AP), β‐actin (ABclonal. AC038), IFN‐γ (Immunoway, YT2279), TNF‐α (Immunoway, YM8306), GZMB (Immunoway, YT6137), METTL7A (Abcam, AB79207; Proteintech, 17092‐1‐AP), B4GALT5 (Affinity, DF3841), PD‐L1 (Proteintech, 17952‐1‐AP), Flag (Proteintech, 66008‐4‐Ig), HA (Proteintech, 51064‐2‐AP), Ubiquitin (PTM Biolabs, PTM‐1107), METTL3 (Proteintech, 15073‐1‐AP), and Myc (Proteintech, 16286‐1‐AP).

### 4.11 Immunoprecipitation and Co‐IP

For Co‐IP, cell lysates were incubated overnight at 4°C with the indicated primary antibodies and Protein A/G magnetic beads (Selleck, B23201). The beads were then washed three times with lysis buffer to remove nonspecifically bound proteins. The immunoprecipitated proteins were eluted and analyzed by immunoblotting with the appropriate antibodies. Immunoprecipitation of HA‑tagged proteins was performed using BeaverBead Anti‑HA Magnetic Beads (BeaverBio, 22205‑T) following the manufacturer's protocol. Briefly, cell lysates were prepared in IP lysis buffer. Anti‑HA magnetic beads were washed twice with 1 × PBS and resuspended. For each 500 µL lysate, 20 µL bead suspension was added, and the mixture was incubated overnight at 4°C on a rotator. After magnetic separation, beads were washed three times with IP lysis buffer. Bound proteins were eluted with 1 × SDS‑PAGE loading buffer at 95°C for 5 min and analyzed by Western Blot.

### 4.12 Immunofluorescence Staining

Cell or tissue slides were fixed with 4% paraformaldehyde. After permeabilization and blocking, slides were incubated overnight at 4°C with the indicated primary antibodies: SLC1A5 (Proteintech, 20350‐1‐AP), METTL7A (ABclonal, A8201), B4GALT5 (Affinity, DF3841), CD8α (Immunoway, YM8067), and GZMB (Immunoway, YT6137), followed by incubation with fluorophore‑conjugated secondary antibodies. Nuclei were counterstained with DAPI (Beyotime Biotechnology, P0131). Images were acquired using a Leica fluorescence microscope.

### 4.13 Immunohistochemistry Staining

Human and mouse GC tissues were fixed in 10% neutral buffered formalin, embedded in paraffin, and sectioned at 4 µm thickness. Following deparaffinization and rehydration, antigen retrieval was performed. After blocking endogenous peroxidase activity and nonspecific binding sites, the sections were incubated overnight at 4°C with primary antibodies: SLC1A5 (Proteintech, 68540‐1‐Ig), METTL7A (ABclonal, 67905‐1‐Ig), B4GALT5 (Zenbio, 823702), CD8α (Immunoway, YM8067), and GZMB (Immunoway, YT6137). Detection was performed using HRP‐conjugated secondary antibody and DAB (Boster, AR1027) as the chromogen. The sections were then counterstained with hematoxylin, dehydrated, cleared, and mounted.

### 4.14 Flow Cytometry

Cell apoptosis was assessed using an Annexin V‐FITC/PI Apoptosis Detection Kit according to the manufacturer's protocol. 5 × 10^5^ cells were collected, washed twice with pre‑cold PBS, and resuspended in 195 µL binding buffer. Then, 5 µL Annexin V‑FITC and 10 µL PI were added, mixed gently, and incubated at room temperature in the dark for 10 min. After adding 200 µL binding buffer, cells were analyzed by flow cytometry. For the analysis of tumor‐infiltrating lymphocytes (TILs), MFC tumors were dissociated into single‐cell suspensions by mechanical mincing followed by enzymatic digestion in RPMI‐1640 medium containing 1 mg/mL Collagenase IV (Solarbio, C8160) and 0.2  mg/mL DNase I (Solarbio, D8077) for 30 min at 37°C. The cells were filtered through 70 µm cell strainers, washed twice with staining buffer (BD Biosciences, 554656). Subsequently, cells were re‐suspended in the staining buffer and labeled with fluorescein‐conjugated antibodies against CD45 (BD Biosciences, 561487), CD3 (BD Biosciences, 553061), CD8α (BD Biosciences, 553035), and GZMB (Invitrogen, 12‐8898‐82). Human PBMC‑derived CD8^+^ T cells were stimulated with Human CD3/CD28 T Cell Activator (Stemcell, 10971) and cultured in ImmunoCult‑XF T Cell Expansion Medium (Stemcell, 10981) supplemented with 10 ng/mL IL‑2 (Stemcell, 78036). Following single or co‑culture, cells were restimulated with Leukocyte Activation Cocktail containing BD GolgiPlug (BD Biosciences, 550583) for 2 h to block intracellular protein transport. Cells were labeled with fluorescein‐conjugated antibodies against CD8 (BD Biosciences, 564116), IFN‐γ (BD Biosciences, 562988), TNF‐α (BD Biosciences, 559321), GZMB (BD Biosciences, 560212), and Ki67 (BD Biosciences, 567719). For intracellular staining, cells were used by Fixation/Permeabilization Kit (BD Biosciences, 554714). For Ki67 staining, cells were used with the Transcription Factor Buffer Set (BD Biosciences, 562574). Dead cells were labeled using the Fixable Viability Stain 520 (BD Biosciences, 565388). Immunostained cells were subsequently analyzed by an Apogee Flow Cytometer. The data were analyzed using FlowJo.

### 4.15 EdU Staining, Colony Formation, and Cell Counting Kit‐8 Assays

The proliferative capacity of GC cells was evaluated using Cell Counting Kit‐8 (CCK‐8; TargetMol, C0005) and EdU staining (Beyotime Biotechnology, C0078) assays. The colony formation assay was performed by seeding 400 cells per well in 6‐well plates. After 10 days of culture, colonies were fixed and stained with crystal violet for quantification. The CCK‐8 assay was used to determine the half‐maximal effective concentration (EC_50_) and the half‐maximal inhibitory concentration (IC_50_) values.

### 4.16 Methylated RNA Immunoprecipitation (MeRIP)‐qPCR

MeRIP‐qPCR was performed to detect m6A enrichment using the Ribobio MeRIP m6A Kit (C11051‐1) according to the manufacturer's instructions. Total RNA was extracted from GC cells and chemically fragmented. The fragmented RNA was then incubated at 4°C with magnetic beads conjugated to an anti‐m6A antibody. After washing, the m6A‐enriched RNA was eluted from the beads and subjected to reverse transcription followed by qPCR analysis. Primers targeting the predicted m6A site in SLC1A5 3′ UTR (identified via the SRAMP database) were used for detection, and their sequences are provided in Table S4.

### 4.17 Dot Blot Assay

Total RNA was extracted using TRIzol reagent. For Dot blot analysis, 125, 250, and 500 ng of mRNA were denatured by heating at 95°C for 5 min, followed by immediate cooling on ice. The denatured samples were then applied to a positively charged nylon membrane and air‐dried. After UV crosslinking, the membrane was blocked and incubated overnight at 4°C with an anti‐m6A primary antibody (Synaptic Systems, 202003). This was followed by a 1 h incubation at room temperature with an HRP‐conjugated anti‐rabbit secondary antibody. Uniform mRNA loading was verified by methylene blue staining (Solarbio, M8030) of the membrane.

### 4.18 Dual‐Luciferase Reporter Assay

Cells were plated in 96‐well plates and transfected with the constructed luciferase reporter plasmids. After 24 h of incubation, luciferase activity was measured using the Dual‐Luciferase Reporter assay detection kit (Beyotime Biotechnology, RG027) following the manufacturer's instructions.

### 4.19 RNA Stability Assay

The stability of SLC1A5 mRNA was assessed by treating cells with actinomycin D (Act‐D; 10 µg/mL; MedChemExpress, HY‐17559) for the indicated durations (0, 2, 4, and 6 h), after which RNA was extracted and analyzed via qRT‐PCR.

### 4.20 Protein Half‐Life Assay

To determine the SLC1A5 protein half‐life, cells were treated with cycloheximide (CHX; 200 µg/mL; MedChemExpress, HY‐12320) for 0, 2, 4, and 8 h, and then subjected to Western blot analysis.

### 4.21 Enzyme‐Linked Immunosorbent Assay (ELISA)

Mouse IFN‐γ, mouse TNF‐α, and mouse GZMB ELISA kits were procured from Ruifan Biotechnology. Standards and serum samples were added to pre‑coated plates, incubated with HRP‑conjugated antibody at 37°C for 60 min, and washed five times. Plates were developed with TMB substrate for 15 min, the reaction was stopped, absorbance was read at 450 nm, and concentrations were calculated from the standard curve.

### 4.22 Isolation of Tumor Interstitial Fluid (TIF)

Tumors were harvested, weighed, washed with ice‑cold PBS, cut into small pieces (5 mm^3^), and placed on 70 µm filters fitted in 50 mL centrifuge tubes. The samples were centrifuged at 500 × g for 15 min at 4°C. The collected TIF was further filtered through 0.22 µm filters for subsequent analysis.

### 4.23 Glutamine Quantification Assay

Glutamine (Gln) levels in tissues, liquid samples, and cells were measured using a colorimetric assay kit (Elabscience, E‐BC‐K853‑M) following the manufacturer's protocol. 0.1 g fresh tissue was homogenized in 0.9 mL 0.9% NaCl, centrifuged at 10 000 × g for 15 min at 4°C, and the supernatant was filtered through a 50 kDa ultrafiltration tube; liquid samples were directly filtered with a 50 kDa ultrafiltration tube; for cells, approximately 1 × 10^6^ cells were lysed in 0.2 mL 0.9% NaCl, centrifuged at 10 000 × g for 15 min at 4°C, and the supernatant was filtered through a 50 kDa ultrafiltration tube. Then, 30 µL of Enzyme Reagent A working solution was added to each well, followed by 50 µL of standard solution or sample; plates were shaken for 3 s and incubated at 37°C for 20 min in the dark. Next, 140 µL of Reaction Working Solution was added, and absorbance at 450 nm (A_1_) was measured. After incubation at 37°C for another 30 min in the dark, absorbance (A_2_) was read, and ΔA was calculated. For co‐culture, all raw glutamine readings were normalized to viable cell counts collected. Specifically, cells were harvested after co‑culture, stained with trypan blue, and viable (unstained) cells were counted using a hemocytometer. Each sample's total glutamine content was divided by its corresponding viable cell number to generate normalized intracellular glutamine concentrations per viable cell.

### 4.24 Nuclear Protein Extraction and m6A Methyltransferase Activity Assay

Nuclear proteins were extracted from GC cells using the EpiQuik Nuclear Extraction Kit I (EpigenTek, OP‐0002) following the manufacturer's instructions. Samples were resuspended in ice‐cold 1× Pre‐Extraction Buffer supplemented with protease inhibitor cocktail and DTT; cytoplasmic fractions were removed after ice incubation, and nuclear pellets were lysed in Extraction Buffer with protease inhibitors and DTT. Nuclear lysates were collected by centrifugation, and m6A methyltransferase activity was measured using the Epigenase m6A Methylase Activity/Inhibition Assay Kit (EpigenTek, P‐9019). Briefly, nuclear extracts were incubated with m6A substrate and SAM‐containing methylase buffer at 37°C. After washing, methylated m6A was detected via capture and detection antibodies, and absorbance at 450 nm was measured to calculate activity against a standard curve.

### 4.25 Statistical Analysis

All statistical analyses were conducted with GraphPad Prism software. Quantitative data are presented as the mean ± standard deviation (SD) from three independent experiments. For comparisons between two independent or paired groups, a two‐tailed unpaired or paired Student′s t‐test was applied, respectively. Comparisons among three or more groups were performed using a one‐way ANOVA. Survival curves were generated using the Kaplan–Meier method and compared by the log‐rank test. Correlations were assessed using Spearman′s rank correlation coefficient. A *p*‐value of less than 0.05 was considered statistically significant, denoted as follows: ^*^ *p* < 0.05, ^**^ *p* < 0.01, ^***^ *p* < 0.001; ns, not significant.

## Author Contributions

MS, SD, and DZ contributed equally to this work. MS, ML, and GL contributed to the design of the work. MS, SD, and DZ performed the experiments. XS, WS, YS, ZC, and TL were responsible for the collection of the clinical samples. MS, SD, LD, and GL analyzed the data and prepared the figures, and wrote the manuscript. JN, QY, ZZ, and JL helped to draft the manuscript. TB and YW contributed to drug screening. All authors read and approved the final manuscript.

## Conflicts of Interest

The authors declare no conflict of interest.

## Supporting information




**Supporting File**: advs76963‐sup‐0001‐SuppMat1.docx.

## Data Availability

The data that support the findings of this study are available from the corresponding author upon reasonable request.
