# Supplementary material for: METTL7A Downregulation Drives SLC1A5‐Mediated Glutamine Competition to Promote Tumor Proliferation and Suppress CD8+ T Cell Immunity in Gastric Cancer
Source: Adv Sci (Weinh). 2026 Aug 3;13(60):e76963. doi: 10.1002/advs.76963 (PMC13430941; doi:10.1002/advs.76963)
Supplement: Supplementary file 1 [file ADVS-13-e76963-s001.docx]

**Supplementary Information**

**METTL7A Downregulation Drives SLC1A5-Mediated Glutamine Competition to Promote Tumor Proliferation and Suppress CD8^+^ T Cell Immunity in Gastric Cancer**

*Mingjun Sun, Shuwei Dang, Dazhi Zhou, Lixian Ding, Xun Sun, Wei Suo, Jirui Niu, Qingzhu Yang, Zhicheng Zhang, Yanyan Sun, Jinxing Li, Zhongsheng Chen, Tao Ban, Yingji Wang, Tianzhu Li, Ming Liu****^*^****, Guodong Li****^*^***

**Supplemental Figure Legends**

**Figure S1. Enrichment Analysis of Hub Genes and Clinical Correlation for SLC1A5 in GC.**

(**A, B**) Functional enrichment analysis of the 30 hub genes, showing the top 10 enriched KEGG pathways (**A**) and Gene Ontology (GO) terms (**B**). (**C–F**) Correlation between SLC1A5 expression and clinicopathological features in 93 GC patients, including AJCC stage (**C**), pathological grade (**D**), tumor invasion depth (**E**), and lymph node metastasis (**F**). (**G, H**) The expression of SLC1A5 in GC tissues compared to adjacent normal tissues from TCGA (**G**) and GSE27342 (**H**). (**I, J**) Kaplan–Meier analysis of the overall survival from GSE14210 (**I**) and GSE22377 (**J**).

**Figure S2. Validation of SLC1A5 cellular models and their glutamine-dependent proliferative phenotypes.**

(**A, B**) Validation of SLC1A5 knockdown efficiency in AGS and MKN-45 cells at the mRNA (**A**) and protein (**B**) levels. n = 3. (**C**) CCK-8 assay in SLC1A5 knockdown AGS and MKN-45 cells. n = 3. (**D–F**) Colony formation (**D**), EdU staining (**E**), and CCK-8 (**F**) assays in AGS and MKN-45 cells cultured in medium containing 2 mM or 4 mM glutamine. n = 3. Scale bars: 100 μm. (**G, H**) Validation of SLC1A5 overexpression efficiency in AGS and MKN-45 cells at the mRNA (**G**) and protein (**H**) levels. n = 3. (**I**) CCK-8 assay of SLC1A5-overexpressing cells under normal (2 mM) or reduced (1 mM) glutamine conditions. n = 3. (**J–L**) Colony formation (**J**), EdU staining (**K**), and CCK-8 (**L**) assays in AGS and MKN-45 cells treated with V-9302. n = 3. Scale bars: 100 μm.

**Figure S3. Glutamine supplementation restores the function of tumor-infiltrating CD8^+^ T cells.**

(**A**) Relative serum levels of GZMB, IFN-γ, and TNF-α measured by ELISA in MFC tumor-bearing BALB/c mice treated with intratumoral PBS or glutamine. n = 5. (**B**) qRT-PCR analysis shows the relative mRNA expression of GZMB, IFN-γ, and TNF-α within MFC tumors from BALB/c mice treated with intratumoral PBS or glutamine. n = 5. (**C**) Western blot analysis shows the relative protein expression of GZMB, IFN-γ, and TNF-α within MFC tumors from BALB/c mice treated with intratumoral PBS or glutamine. n = 5. (**D**) Relative glutamine concentration in paired human GC and adjacent normal tissues. n = 5. (**E**) Relative glutamine levels in plasma and TIF collected from subcutaneous GC xenograft-bearing mice. n = 5. (**F**) Relative intracellular glutamine concentrations in MKN-45 cells cultured with or without CD8⁺ T cells, and CD8⁺ T cells cultured with or without MKN-45 cells under the specified experimental conditions. n = 3. (**G**) Flow cytometric analysis of human CD8^+^ T cells positive for Ki67. n = 3.

**Figure S4. Functional and cellular characterization of SLC1A5 in the GC microenvironment.**

(**A**) Canonical marker genes expression across identified cell types. (**B, C**) t-SNE plots of SLC1A5 expression in cells from normal tissues **(B)** and tumor tissues **(C)**. (**D**) Spatial transcriptomic analysis of SLC1A5 expression in paired human GC and adjacent normal tissues. (**E**) The number of predicted interactions among different cell types. (**F**) Western blot analysis comparing SLC1A5 protein expression between human GC cell lines (AGS, MKN-45) and activated human CD8⁺ T cells. (**G, H**) Viability of MKN-45 cells assessed by LDH release (**G**) and crystal violet staining (**H**) assays under conditions including culture alone or co-culture with sh-NC or sh-SLC1A5 MKN-45 cells, with or without glutamine supplementation. n = 3. (**I, J**) Validation of SLC1A5 knockdown efficiency in MFC cells at the mRNA (**I**) and protein (**J**) levels. n = 3. (**K**) Representative H&E staining of tumor sections from mice under the specified experimental conditions. Scale bars: 100 μm. (**L**) Relative serum levels of GZMB, IFN-γ, and TNF-α measured by ELISA under the specified experimental conditions. n = 5. (**M**) Representative immunofluorescence staining images of tumor sections from mice under the specified experimental conditions. Scale bars: 50 μm.

**Figure S5. METTL7A deficiency upregulates SLC1A5 expression in GC.**

(**A**) Correlation analysis between METTL7A and SLC1A5 IHC scores in the in-house GC cohort. (**B–E**) Association between METTL7A expression and clinicopathological features, including AJCC stage (**B**), pathological grade (**C**), invasion depth (**D**), lymph node metastasis (**E**). (**F, G**) The expression of METTL7A in GC tissues compared to adjacent normal tissues from TCGA (**F**) and GSE27342 (**G**). (**H**) Kaplan–Meier survival analysis of METTL7A expression from GSE27342. (**I, J**) Validation of METTL7A overexpression efficiency in AGS and MKN-45 cells at the mRNA (**I**) and protein (**J**) levels. n = 3. (**K–M**) Western blot (**K**), qRT-PCR (**L**), and immunofluorescence staining (**M**) analysis of SLC1A5 in METTL7A-overexpressing cells. Scale bars: 5 μm. n = 3. (**N**) Western blot analysis of METTL7A and SLC1A5 protein levels in Jurkat T cells with or without METTL7A knockdown. (**O**) CCK-8 assay showing the proliferation of AGS and MKN-45 cells under the indicated conditions. n = 3. (**P, Q**) Viability of MKN-45 cells assessed by LDH release (**P**) and crystal violet staining (**Q**) assays under the indicated conditions. n = 3. (**R**) Relative m6A methyltransferase activity in GC cells with or without METTL7A overexpression. n = 3. (**S, T**) qRT-PCR (**S**) and Western blot (**T**) analysis of METTL3 in METTL7A-overexpressing cells. n = 3. (**U**) MeRIP-qPCR analysis of m6A modification on SLC1A5 mRNA in GC cells with or without METTL7A overexpression. n = 3. (**V**) Schematic of the primers sets used for 3′ UTR-specific MeRIP-qPCR.

**Figure S6. Interaction of B4GALT5 with SLC1A5 and its prognostic significance in GC.**

(**A**) qRT-PCR analysis validating the knockdown efficiency of six candidate N-glycosyltransferases in AGS and MKN-45 cells. n = 3. (**B**) Correlation analysis between B4GALT5 and SLC1A5 mRNA expression from TCGA. (**C, D**) Validation of B4GALT5 overexpression efficiency in AGS and MKN-45 cells at the mRNA (**C**) and protein (**D**) levels. n = 3. (**E, F**) Molecular docking model predicting the interaction between B4GALT5 and SLC1A5. (**G**) qRT-PCR analysis of B4GALT5 mRNA expression in paired GC and adjacent noncancerous tissues. (**H**) Western blot analysis of B4GALT5 protein levels in paired GC and adjacent noncancerous tissues. (**I**) qRT-PCR analyzing B4GALT5 mRNA in GES-1 and GC cells. (**J**) Western blot analyzing B4GALT5 protein in GES-1 and GC cells. (**K**) Representative IHC images of B4GALT5 protein expression in paired GC and adjacent noncancerous tissues. Scale bar: 50 µm. (**L, M**) The expression of B4GALT5 in GC tissues compared to adjacent normal tissues from TCGA (**L**) and GSE27342 (**M**). (**N**) Kaplan–Meier survival analysis of B4GALT5 expression from GSE27342. (**O**) Western blot analysis comparing B4GALT5 and METTL7A protein expression between human GC cell lines (AGS, MKN-45) and activated human CD8⁺ T cells.

**Figure S7. The SLC1A5 protein half-life in AGS cells with or without METTL7A overexpression.**

(**A**) CHX chase assay assessed by Western blot to determine SLC1A5 protein half-life in GC cells with or without METTL7A overexpression. (**B**) Western blot analysis of SLC1A5 N-glycosylation in HEK293T cells co-transfected with WT or N163Q mutant SLC1A5 and either full-length (FL) B4GALT5.

**Figure S8. The inhibitory effects, toxicity, and mechanisms of Luteolin in GC.**

(**A**) Detailed view of the predicted interaction interface between Lut and key amino acid residues of METTL7A. (**B, C**) qRT-PCR (**B**) and Western blot (**C**) analysis of METTL7A knockdown efficiency. n = 3. (**D, E**) The protein (**D**) and mRNA (**E**) levels of METTL7A, B4GALT5, and SLC1A5 under the indicated conditions. n = 3. (**F, G**) Cell viability assessed by Colony formation (**F**) and EdU staining (**G**) assays in AGS and MKN-45 cells treated with increasing concentrations of Lut for 24 h. Scale bars: 100 μm. n = 3. (**H–J**) Representative images (**H**), tumor growth curves (**I**), and tumor weights (**J**) in MFC xenograft-bearing mice across different treatment groups. n = 5. (**K**) Representative H&E staining images of major organs (heart, liver, spleen, lungs, kidneys) from mice. (**L**) The protein levels of METTL7A, B4GALT5, and SLC1A5 under the indicated conditions. (**M, N**) qRT-PCR (**M**) and Western blot (**N**) analysis of METTL7A overexpression efficiency in MFC cells. n = 3. (**O–Q**) Representative images (**O**), tumor growth curves (**P**), and tumor weights (**Q**) in MFC xenograft-bearing mice across different treatment groups. n = 5. (**R**) The protein levels of PD-L1 in AGS and MKN-45 cells with Lut treatment. (**S**) Relative serum levels of GZMB, IFN-γ, and TNF-α measured by ELISA under the specified experimental conditions. n = 5. (**T**) Representative immunofluorescence staining images of tumor sections from mice under the specified experimental conditions. Scale bars: 50 μm.

**Table S1. Sequences of shRNA**

| SLC1A5-human-1 | 5'-GCTTGGTAGTGTTTGCCATCG-3' |
| --- | --- |
| SLC1A5-human-2 | 5'-GGATGTGGGTTTACTCTTTGC-3' |
| SLC1A5-human-3 | 5'-GCTGATGATGAAGTGCGTGGA-3' |
| SLC1A5-mouse-1 | 5'-CTTCATCAGCCTCGGCAAATA-3' |
| SLC1A5-mouse-2 | 5'-GTATCCAGCGGGAGATCAATT-3' |
| SLC1A5-mouse-3 | 5'-TAGATCTCGTGAGGAATATTT-3' |

**Table S2. Sequences of siRNA**

| si-METTL7A | sense sequence | 5'-GCAGCUGAGUGUUCGACUUTT-3' |
| --- | --- | --- |
|  | antisense sequence | 5'-AAGUCGAACACUCAGCUGCTT-3' |
| si-UGT1A6 | sense sequence | 5'-GGAGCAUACAUUCAGCAGATT-3' |
|  | antisense sequence | 5'-UCUGCUGAAUGUAUGCUCCTT-3' |
| si-B4GALT5 | sense sequence | 5'-CCAGUUUCUUGGAAGGUAUTT-3' |
|  | antisense sequence | 5'-AUACCUUCCAAGAAACUGGTT-3' |
| si-B4GALT1 | sense sequence | 5'-CCAUGCUGAUUGAGUUUAATT-3' |
|  | antisense sequence | 5'-UUAAACUCAAUCAGCAUGGTT-3' |
| si-B3GNT3 | sense sequence | 5'-CAGACAACAUGGUCUUCUATT-3' |
|  | antisense sequence | 5'-UAGAAGACCAUGUUGUCUGTG-3' |
| si-ST6GALNAC3 | sense sequence | 5'-GGCAAUGGCAUCGUUUACATT-3' |
|  | antisense sequence | 5'-UGUAAACGAUGCCAUUGCCTT-3' |
| si-EXT2 | sense sequence | 5'-CCAUCUCCCGGGAGUAUAATT-3' |
|  | antisense sequence | 5'-UUAUACUCCCGGGAGAUGGTT-3' |

**Table S3. Sequences of Primers**

| SLC1A5 | Forward primer | 5'-TTTTTCCTGGTCACCACGCT-3' |
| --- | --- | --- |
|  | Reverse primer | 5'-TGGAAGGGAAGATATTTCTCGCAA-3' |
| METTL7A | Forward primer | 5'-GCTGAACTTTCTGGGCTTGTG-3' |
|  | Reverse primer | 5'-TTGCTTGCCATCTGTTCGTTG-3' |
| β-actin | Forward primer | 5'-AACTGGGACGACATGGAGAAAA-3' |
|  | Reverse primer | 5'-GGATAGCACAGCCTGGATAGCA-3' |
| UGT1A6 | Forward primer | 5'-GGGACACCCTGAACTTCTTT-3' |
|  | Reverse primer | 5'-ACAGATGGTAGGCCCAAATAC-3' |
| EXT2 | Forward primer | 5'-GTGTGCGTCGGTCAAGTATAA-3' |
|  | Reverse primer | 5'-GACAATGGAGAAGAGGGTGATATAG-3' |
| ALG9 | Forward primer | 5'-GACCGAGTTATCTGGGAACAAA-3' |
|  | Reverse primer | 5'-CTCAGGAGAGCAGCACATAAC-3' |
| ST6GALNAC3 | Forward primer | 5'-CTGGTTGTGCGTCTTGTAAATG-3' |
|  | Reverse primer | 5'-GCCTGTATGTGTAGGAGAATGG-3' |
| B4GALT1 | Forward primer | 5'-GGCAAAGCAGAACCCAAATG-3' |
|  | Reverse primer | 5'-GTTGCGGAATGGAATGATGATG-3' |
| UGGT1 | Forward primer | 5'-CCTGTTTACCTCTCTGGCTATG-3' |
|  | Reverse primer | 5'-GTGGTGTTTACCTCAGTTCCT-3' |
| STT3A | Forward primer | 5'-CGTTTCTCTCACCGGATCTATG-3' |
|  | Reverse primer | 5'-GATGAAAGGACAGGCTGGAA-3' |
| ALG1 | Forward primer | 5'-GATGGACACAACCTTCCTTCTC-3' |
|  | Reverse primer | 5'-GTGGATGAGGCGGCTATAATAC-3' |
| MGAT2 | Forward primer | 5'-CCTAACGAGTTTCCAGGTAGTG-3' |
|  | Reverse primer | 5'-GGGAGAATTTGGCCTCTCTATAA-3' |
| B4GALT5 | Forward primer | 5'-TCTTTTCTCTCTCGTCCTCGCT-3' |
|  | Reverse primer | 5'-GCACCGATTGTTCTCACGTTG-3' |
| B3GALT6 | Forward primer | 5'-GCGCTTCGACACCGAATA-3' |
|  | Reverse primer | 5'-GCTTCTCCAGCATGTCCTG-3' |
| ALG3 | Forward primer | 5'-CACCCAACCAGATCGTTTCTA-3' |
|  | Reverse primer | 5'-GTAGGGCAGTGTGTGGAAATA-3' |
| B3GNT3 | Forward primer | 5'-TCTTCAACCTCACGCTCAAG-3' |
|  | Reverse primer | 5'-GTGTGCAAAGACGTCATCATC-3' |
| ALG5 | Forward primer | 5'-CAGCGTTCTTACTTCCGTACTC-3' |
|  | Reverse primer | 5'-CTGTGTGTCCCTGATTCCTTT-3' |
| STT3B | Forward primer | 5'-TTGATGATAACCGCTGGCCT-3' |
|  | Reverse primer | 5'-AAGTCCTGCTCCTTGGTTCC-3' |
| B3GAT3 | Forward primer | 5'-TGCTGACGATGACAACACCT-3' |
|  | Reverse primer | 5'-CTGTGTGGAAGCCCACTACC-3' |
| ALG2 | Forward primer | 5'-CCTGCTGCTCTCCATCAACA-3' |
|  | Reverse primer | 5'-GTCATAACCACCTGCCACGA-3' |
| METTL3 | Forward primer | 5'-TTGTCTCCAACCTTCCGTAGT-3' |
|  | Reverse primer | 5'-CCAGATCAGAGAGGTGGTGTAG-3' |
| SLC1A5 (mouse) | Forward primer | 5'-ATCCAGCGGGAGATCAATTC-3' |
|  | Reverse primer | 5'-AGCCACACCAAAGACGATAG-3' |
| β-actin (mouse) | Forward primer | 5'-CCTTCCAGCAGATGTGGATCA-3' |
|  | Reverse primer | 5'-TCAGTAACAGTCCGCCTAGA-3' |
| IFN-γ (mouse) | Forward primer | 5'-CTCTTCCTCATGGCTGTTTCT-3' |
|  | Reverse primer | 5'-TTCTTCCACATCTATGCCACTT-3' |
| TNF-α (mouse) | Forward primer | 5'-TTGTCTACTCCCAGGTTCTCT-3' |
|  | Reverse primer | 5'-GAGGTTGACTTTCTCCTGGTATG-3' |
| GZMB (mouse) | Forward primer | 5'-GGCGCAATGTCAATGTGAAG-3' |
|  | Reverse primer | 5'-CTGTCAGCTCAACCTCTTGTAG-3' |
| METTL7A (mouse) | Forward primer | 5'-GCTGAACCTTCTAGGCTTGT-3' |
|  | Reverse primer | 5'-CTCGCCATCTGCTCATTGTA-3' |

**Table S4. MeRIP-qPCR primers for the SLC1A5 m6A sites**

| Primer 1 | Forward primer | 5'-AGGAAACCCCCTCCTCAAAC-3' |
| --- | --- | --- |
|  | Reverse primer | 5'-CCAGTGTCCAAAGAGCACCC-3' |
| Primer 2 | Forward primer | 5'-CAGGACAGGAGATCTGGGATG-3' |
|  | Reverse primer | 5'-CATGAGTGAGAACTGGGGGTT-3' |
| Primer 3 | Forward primer | 5'-CTGTGACCTCCTGTCCCCAT-3' |
|  | Reverse primer | 5'-ACAGCAGGTATTTGTCCTCAGC-3' |
